# Physiological and Transcriptomic Insights into Melatonin-Mediated Alleviation of Cadmium Stress in the Endangered Plant *Plantago fengdouensis*

**DOI:** 10.3390/plants15203143

**Published:** 2026-10-14

**Authors:** Yang Peng, Bin-Ru Wang, Jia-Bin Zhang, You-Wei Zuo, Xin Liang, Sheng-Mao Zhou, Shi-Qi You, Hong-Ping Deng

**Affiliations:** Key Laboratory of Eco-Environment in the Three Gorges Reservoir Region, Ministry of Education, School of Life Sciences, Southwest University, Beibei, Chongqing 400715, China

**Keywords:** endangered plants, *Plantago fengdouensis*, cadmium stress, melatonin, physiological characteristics, transcriptomics

## Abstract

*Plantago fengdouensis* is an endangered species threatened by habitat loss, population fragmentation, and cadmium (Cd) contamination. This study evaluated the effects of exogenous melatonin (MT) on Cd tolerance from seed germination to the seedling stage. In Petri-dish assays, 50–100 mg L^−1^ Cd markedly inhibited germination; 50 mg L^−1^ was selected as the lowest concentration producing severe inhibition while retaining sufficient germination for mitigation testing, and 100 μM MT provided the strongest alleviation. In pot experiments, MT promoted seedling growth, shoot and root biomass accumulation, and root and leaf development while reducing Cd accumulation and root-to-shoot translocation. Physiological analyses showed that 200–250 μM MT most effectively alleviated Cd-associated damage by maintaining photosynthesis, enhancing antioxidant and osmotic-adjustment responses, and modulating endogenous hormones. Transcriptome analysis identified 4871 differentially expressed genes (DEGs) between C50M200 and C50M0, with prominent enrichment in plant hormone signaling, cell-wall/plasma-membrane functions, secondary metabolism, and photosynthesis. Exploratory weighted gene co-expression network analysis (WGCNA) further identified treatment-associated modules and candidate hub genes. Overall, the data support coordinated MT-associated responses involving stronger antioxidant defense, reduced Cd accumulation/translocation, and hormone-signaling remodeling. Because transcriptomes were obtained from leaves and WGCNA included nine samples, the inferred molecular mechanisms should be regarded as candidate pathways pending targeted expression and functional validation.

## 1. Introduction

*Plantago fengdouensis* (*P. fengdouensis*) is a perennial herb of the *Plantago* genus in the Plantaginaceae family, and it is an endangered species unique to the Three Gorges Reservoir Area. Its wild distribution is extremely limited, with fragmented habitats and scarce individual populations, remaining in a long-term endangered state. It has been assessed as Endangered (EN) in the “China Biodiversity Red List (2020)” and is also listed as a nationally protected wild plant under the second-level protection [1].

Cadmium (Cd) is a non-essential heavy metal that is taken up predominantly by roots through apoplastic binding and transporter-mediated symplastic pathways, with subsequent distribution controlled by root sequestration and long-distance transport processes [2,3,4]. Studies in rice have identified transport components such as OsLCT1, OsNramp5, and OsHMA3 that influence Cd uptake, sequestration, and allocation [3,5,6]. At the cellular and whole-plant levels, Cd can damage organelles, inhibit cell division and root growth, impair photosynthesis, and induce reactive oxygen species (ROS) accumulation and membrane lipid peroxidation [7,8,9,10,11,12,13,14]. Plant acclimation involves antioxidant and osmotic-adjustment systems together with transcriptional, MAPK, hormonal, transporter, and epigenetic regulation [15,16,17,18]. These responses are particularly relevant to narrowly distributed endangered plants, for which additional abiotic stress can further constrain establishment and population recovery.

Melatonin (MT) is an endogenous indoleamine that can enhance plant tolerance to heavy metals through multiple, dose- and species-dependent processes. Appropriate MT concentrations can reduce Cd accumulation or root-to-shoot translocation by modulating metal transport, cell-wall binding, sulfur metabolism, and vacuolar sequestration [19,20,21,22,23]. MT treatment has also been associated with maintenance of photosynthetic pigments and photochemical performance, enhanced enzymatic and non-enzymatic antioxidant capacity, and lower oxidative-damage markers under Cd stress [24,25,26]. In addition, MT interacts with auxin, gibberellin, abscisic acid, cytokinin, and other signaling pathways and can reshape carbon-nitrogen and secondary metabolism [27,28,29,30]. Rather than assuming a universal mechanism, these findings indicate that the protective response to MT must be evaluated in a species- and concentration-specific context, which is especially important for *P. fengdouensis*.

Therefore, this study aims to investigate the response mechanisms of *P. fengdouensis* under Cd stress when treated with exogenous MT. Specifically, the main objectives are: (1) To reveal the alleviating effects of exogenous MT on seed germination in *P. fengdouensis* under Cd stress; (2) To determine the impacts of exogenous MT on the morphological structure and Cd uptake of seedlings under Cd stress; (3) To identify the physiological and biochemical effects of exogenous MT on seedlings under Cd stress; (4) To evaluate the molecular regulatory networks involved in the effects of exogenous MT on *P. fengdouensis* under Cd stress. To address these questions, we investigated the morphological, physiological, and transcriptional responses of *P. fengdouensis* under heavy metal Cd stress at different MT concentrations.

## 2. Result

### 2.1. Effects of Exogenous MT on Seed Germination and Early Seedling Development of P. fengdouensis Under Cd Stress

**Figure 1:**
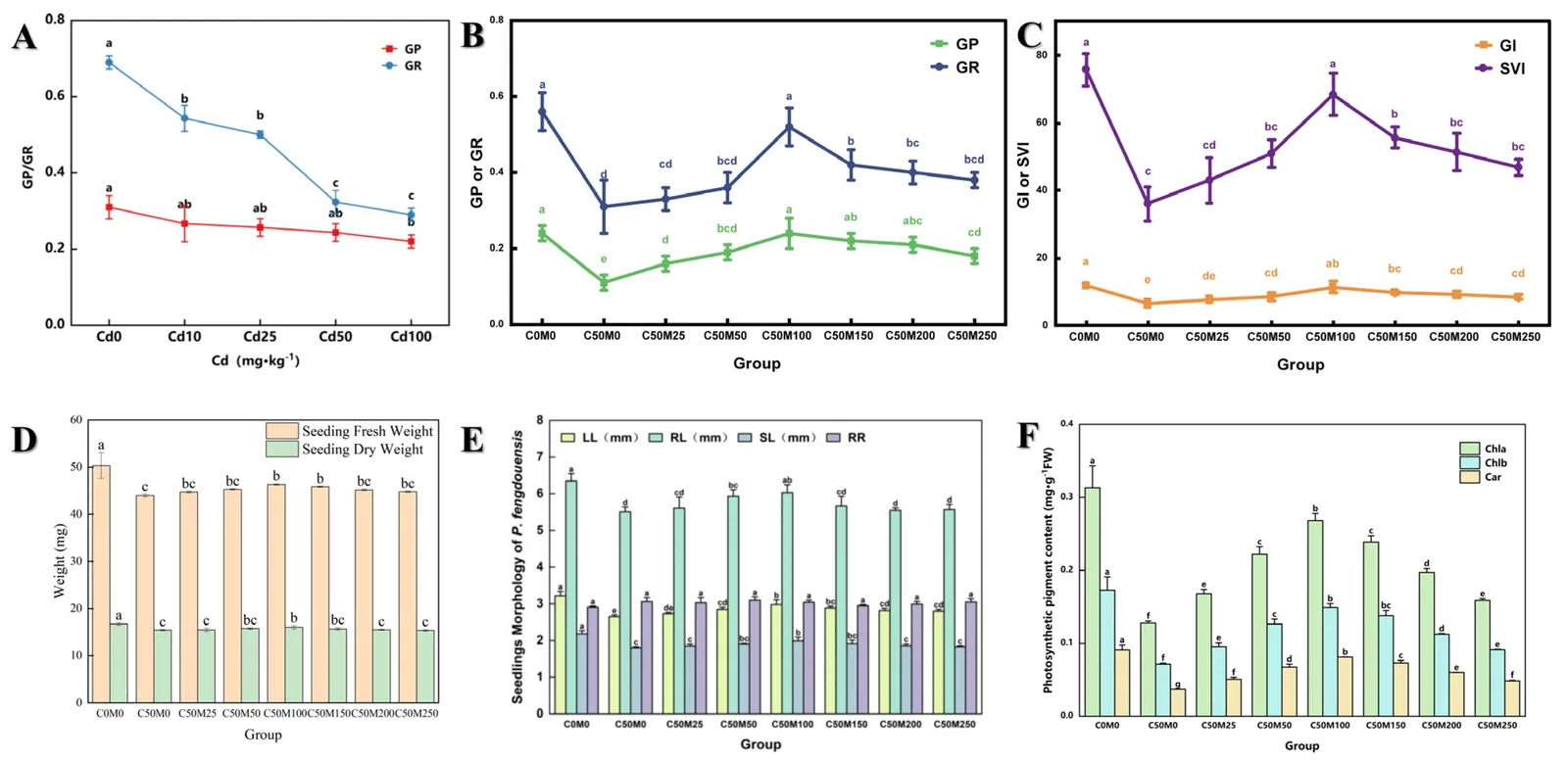
Effects of Cd and exogenous melatonin (MT) on seed germination and early seedling development of *Plantago fengdouensis*. (**A**): Germination potential (GP) and germination rate (GR) under 0, 10, 25, 50, and 100 mg L^−1^ Cd; (**B**–**F**): responses to 0–250 μM MT under 50 mg L^−1^ Cd; (**B**): GP and GR; (**C**): germination index (GI) and seed vigor index (SVI); (**D**): seedling fresh and dry biomass; (**E**): leaf length (LL), root length (RL), stem length (SL), and root-to-shoot length ratio (RR); (**F**): chlorophyll a (Chl a), chlorophyll b (Chl b), and carotenoid (Car) contents in early seedlings.; Different lowercase letters above the data points of line graphs or the tops of bars in bar graphs indicate significant differences among different treatment groups for the same indicator (*p* < 0.05). The same below.

Cd stress significantly inhibited seed germination and early seedling development of *P. fengdouensis* (Figure 1). Increasing Cd concentrations (25–100 mg L^−1^) significantly reduced germination potential and germination rate relative to C0 (*p* < 0.05), and germination was lowest at 100 mg L^−1^ Cd (57.91% lower than the control). Because 50 and 100 mg L^−1^ Cd produced statistically similar inhibition (*p* > 0.05), 50 mg L^−1^ was selected for the MT mitigation experiment as the lower concentration that caused strong stress while retaining sufficient germination for evaluating recovery. Under 50 mg L^−1^ Cd, the germination potential, germination rate, germination index, and seed vigor index decreased by 54.17%, 44.64%, 44.94%, and 52.34%, respectively, relative to C0M0. MT alleviated these effects, with 100 μM producing the strongest response: the four germination indices increased by 118.18%, 67.74%, 72.80%, and 89.11%, respectively, relative to C50M0, and were not significantly different from C0M0 (*p* > 0.05) (Figure 1B,C). Cd also reduced seedling fresh and dry biomass by 12.69% and 7.72%, respectively, whereas 100 μM MT increased them by 5.39% and 3.96% relative to C50M0 (Figure 1D). Root, stem, and leaf lengths were reduced by Cd and partially restored by MT (Figure 1E). Photosynthetic pigment contents were strongly reduced by Cd but increased after MT treatment, with the largest recovery around 100 μM MT (Figure 1F). These results identify 100 μM MT as the most effective concentration for the germination/early-seedling assay.

### 2.2. Effects of Exogenous MT on Growth, Morphology, Anatomy, and Cd Accumulation of P. fengdouensis Seedlings

**Figure 2:**
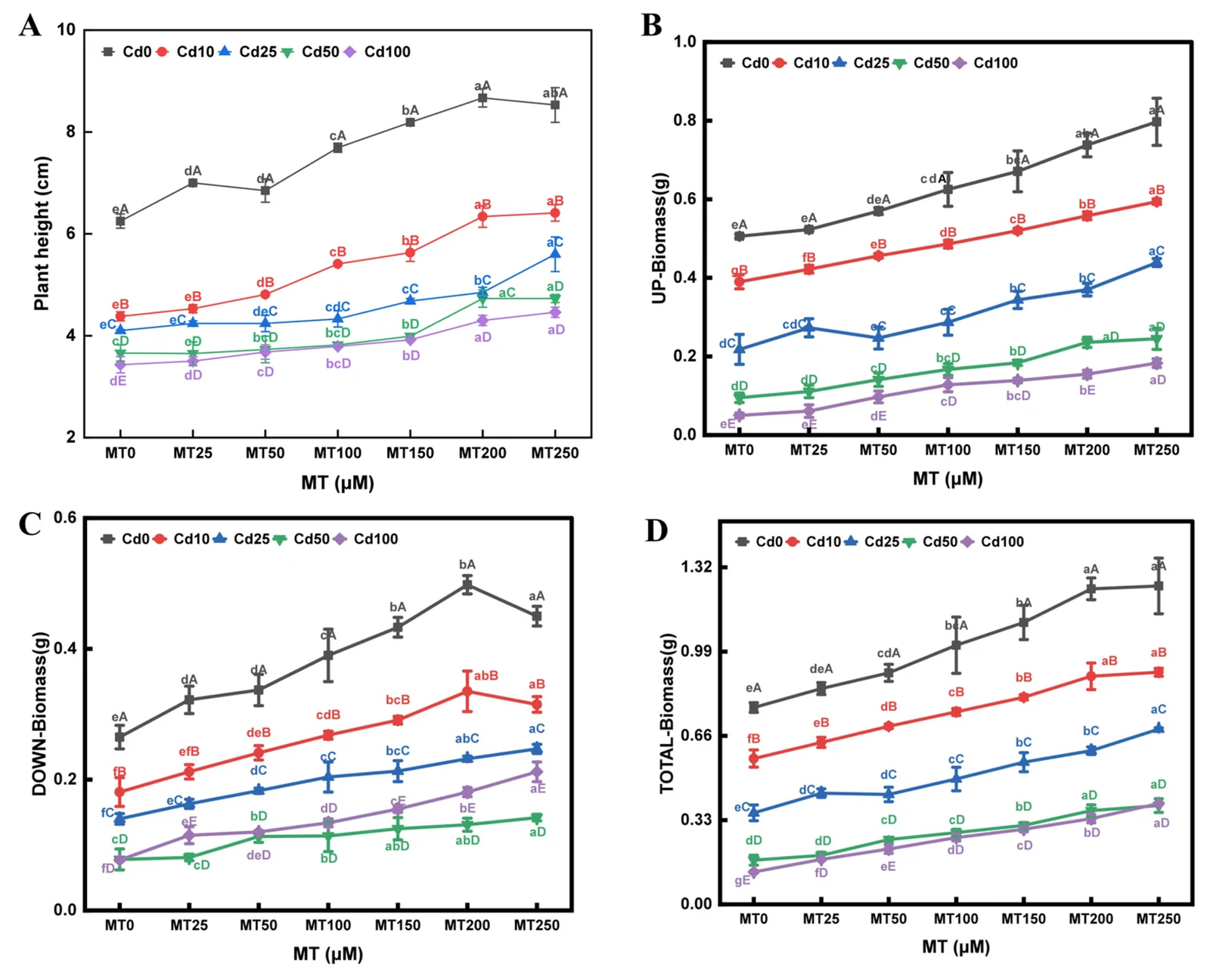
Effects of exogenous MT on growth and biomass of four-month-old *P. fengdouensis* seedlings after 15 d of Cd/MT treatment. (**A**): Plant height; (**B**): shoot biomass; (**C**): root biomass; (**D**): total biomass. Cd0, Cd10, Cd25, Cd50, and Cd100 denote 0, 10, 25, 50, and 100 mg Cd kg^−1^ dry substrate, respectively; MT0, MT25, MT50, MT100, MT150, MT200, and MT250 denote 0, 25, 50, 100, 150, 200, and 250 μM MT, respectively. Different uppercase letters indicate significant differences (*p* < 0.05) among Cd concentrations at the same MT concentration, and different lowercase letters indicate significant differences among MT concentrations at the same Cd concentration.

Four-month-old, uniformly developed *P. fengdouensis* seedlings were subjected to the Cd × MT treatments for 15 d. Seedling height decreased with increasing Cd concentration (Figure 2A); at 100 mg kg^−1^ Cd (dry-substrate basis), height was 45.20% lower than C0M0, with no significant difference between 50 and 100 mg kg^−1^ Cd. MT significantly alleviated growth inhibition. In the absence of Cd, 200 μM MT produced the greatest height (8.67 cm, +38.64% relative to C0M0), whereas under 10 mg kg^−1^ Cd, 250 μM MT yielded 6.41 cm. Across most Cd levels, 200 and 250 μM MT did not differ significantly. Cd progressively reduced shoot, root, and total biomass, whereas MT increased biomass across Cd treatments (Figure 2B–D). At 0 mg kg^−1^ Cd, 250 μM MT maximized shoot biomass (0.80 g) and total biomass (1.25 g), with values not significantly different from 200 μM MT. Under 10 mg kg^−1^ Cd, 250 μM MT produced the strongest biomass recovery. Overall, 200–250 μM MT most consistently alleviated Cd-associated suppression of seedling growth and biomass.

**Figure 3:**
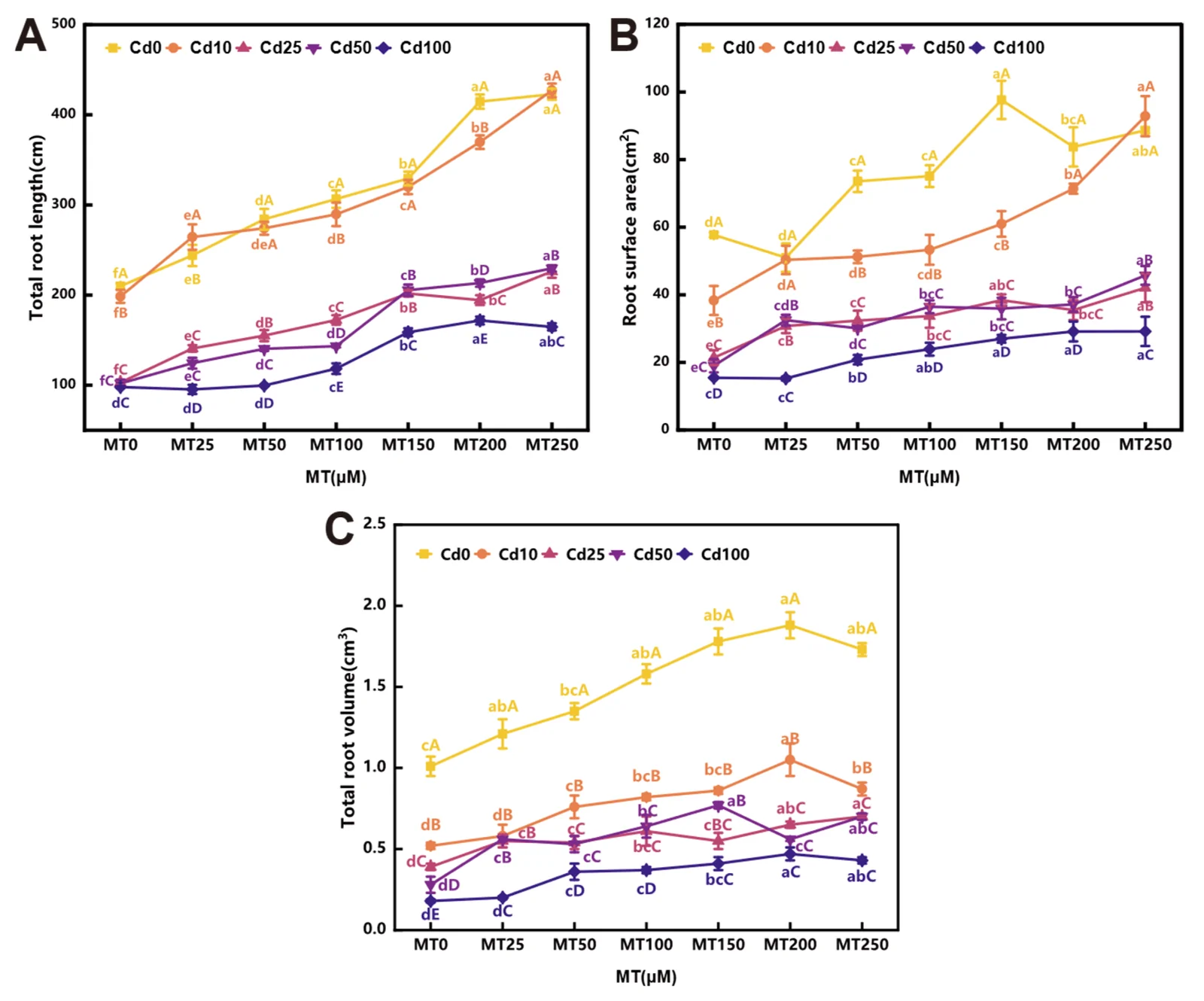
Effects of exogenous MT on root morphological traits of *P. fengdouensis* seedlings under Cd stress. (**A**): Total root length; (**B**): Root surface area; (**C**): Total root volume. Note: Cd0, Cd10, Cd25, Cd50, and Cd100 represent cadmium stress concentrations of 0, 10, 25, 50, and 100 mg·kg^−1^, respectively; MT0, MT25, MT50, MT100, MT150, MT200, and MT250 represent exogenous melatonin concentrations of 0, 25, 50, 100, 150, 200, and 250 μM, respectively. Different uppercase letters indicate significant differences (*p* < 0.05) among cadmium stress concentrations under the same MT concentration, while different lowercase letters denote significant differences (*p* < 0.05) among MT concentrations under the same cadmium stress concentration.

**Figure 4:**
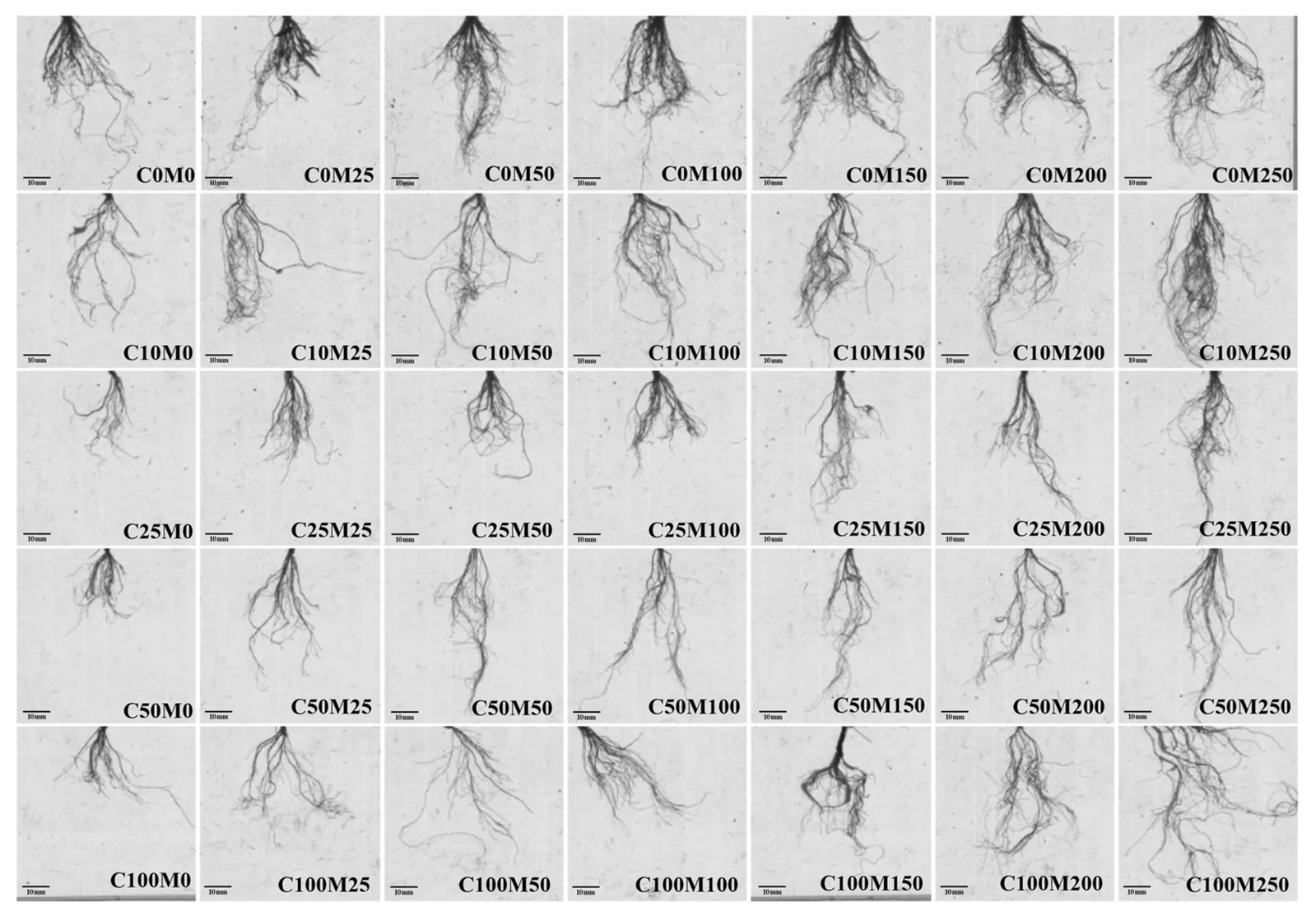
Root scanning images of *P. fengdouensis* seedlings under combined exogenous MT and Cd stress. Note: A total of 35 groups were treated with combinations of cadmium stress and exogenous melatonin. Rows 1 to 5 represent 0, 10, 25, 50, and 100 mg·kg^−1^ Cd, respectively; columns 1 to 7 represent 0, 25, 50, 100, 150, 200, and 250 μM exogenous MT, respectively.

Cd stress inhibited root growth in *P. fengdouensis* seedlings, with inhibition positively correlated to Cd concentration. At 100 mg/kg Cd, root length, surface area, and volume decreased by 53.33%, 73.25%, and 82.18%, respectively; at 50 mg/kg Cd, reductions were 51.41%, 67.05%, and 72.28%, respectively, with no significant difference in root length between concentrations. Exogenous MT promoted root growth concentration-dependently. Optimal MT levels varied by parameter and Cd level: for length, 250 μM at 0–50 mg/kg Cd and 200 μM at 100 mg/kg Cd; for surface area, 250 μM at 10–100 mg/kg Cd and 150 μM at 0 mg/kg Cd; for volume, 200 μM at 0–10 mg/kg Cd, 250 μM at 25 mg/kg Cd, 150 μM at 50 mg/kg Cd, and 200 μM at 100 mg/kg Cd. Overall, Cd stress inhibited root growth, and exogenous MT alleviated this effect, especially at 150–250 μM (Figure 3A–C). Representative root scanning images of the 35 Cd × MT treatment combinations are shown in Figure 4.

**Figure 5:**
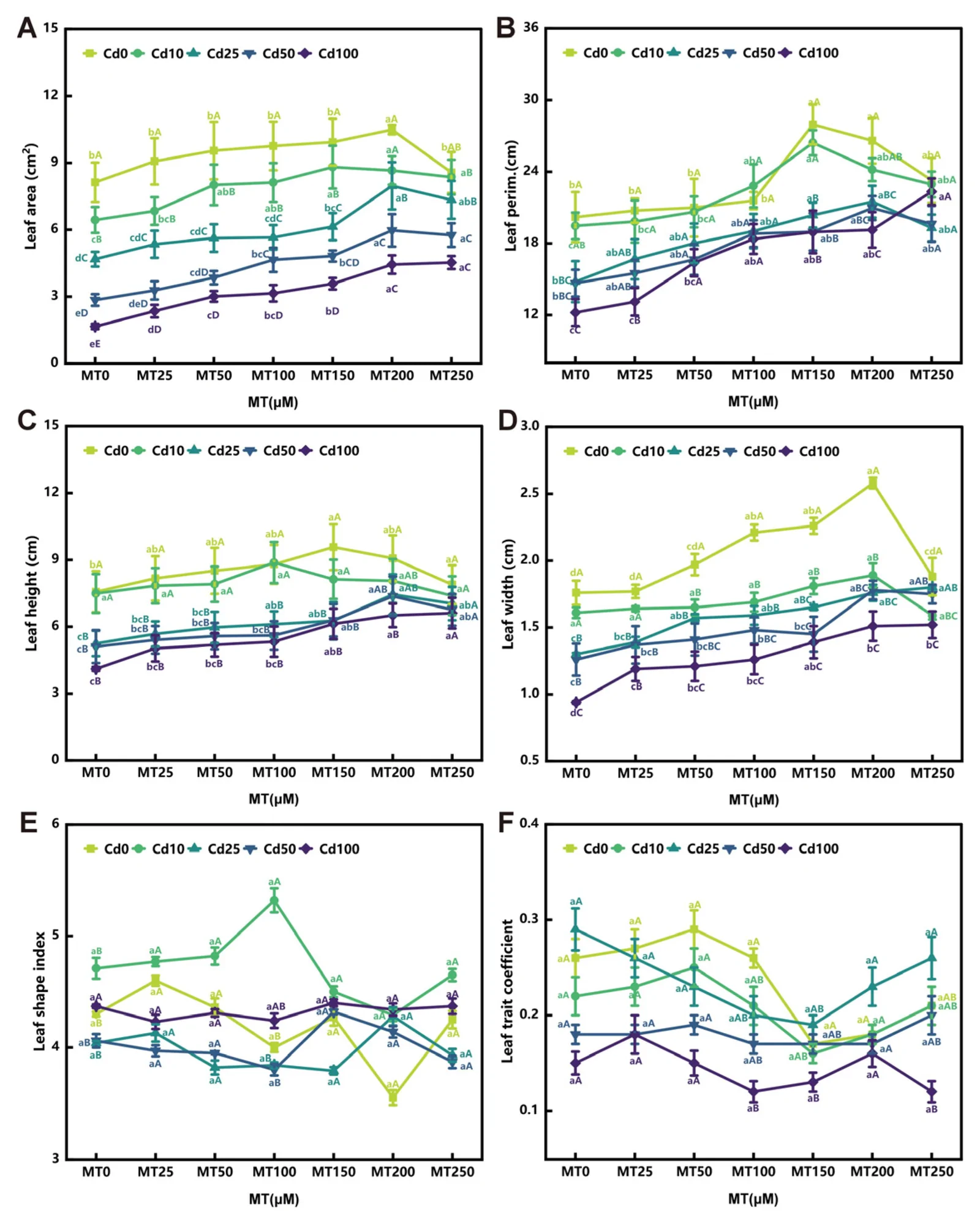
Effects of exogenous MT on leaf morphological traits of *P. fengdouensis* seedlings under Cd stress. (**A**): Leaf area; (**B**): Leaf perimeter; (**C**): Leaf length; (**D**): Leaf width; (**E**): Leaf shape index; (**F**): Leaf trait index. Note: Cd0, Cd10, Cd25, Cd50, and Cd100 represent cadmium stress concentrations of 0, 10, 25, 50, and 100 mg·kg^−1^, respectively; MT0, MT25, MT50, MT100, MT150, MT200, and MT250 represent exogenous melatonin concentrations of 0, 25, 50, 100, 150, 200, and 250 μM, respectively. Different uppercase letters indicate significant differences (*p* < 0.05) among cadmium stress concentrations under the same MT concentration, while different lowercase letters denote significant differences (*p* < 0.05) among MT concentrations under the same cadmium stress concentration.

**Figure 6:**
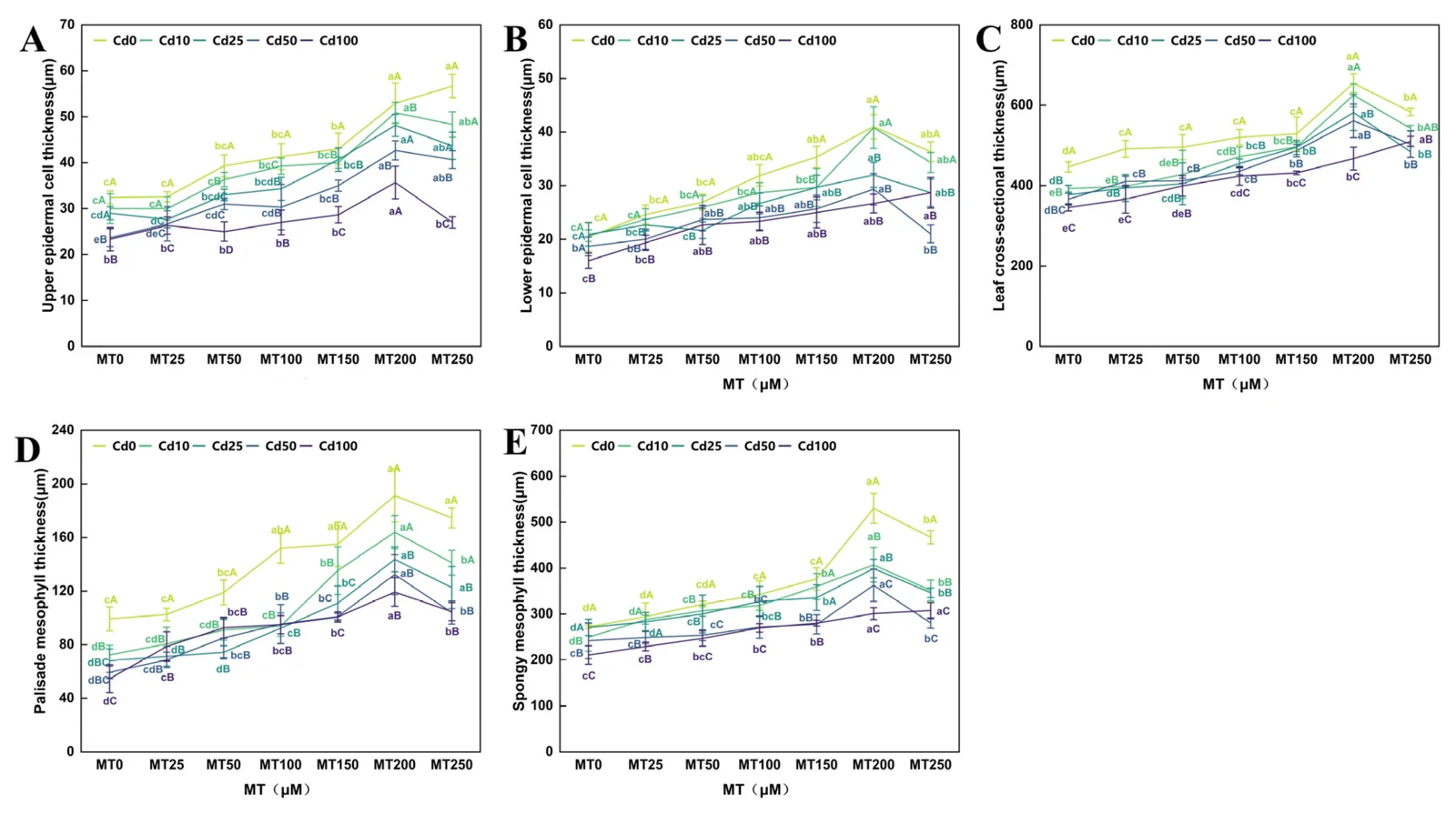
Effects of exogenous MT on leaf anatomical characteristics of *P. fengdouensis* seedlings under Cd stress. (**A**): Upper epidermal cell thickness; (**B**): Lower epidermal cell thickness; (**C**): Leaf cross-sectional thickness; (**D**): Palisade mesophyll thickness; (**E**): Spongy mesophyll thickness. Note: Cd0, Cd10, Cd25, Cd50, and Cd100 represent cadmium stress concentrations of 0, 10, 25, 50, and 100 mg·kg^−1^, respectively; MT0, MT25, MT50, MT100, MT150, MT200, and MT250 represent exogenous melatonin concentrations of 0, 25, 50, 100, 150, 200, and 250 μM, respectively. Different uppercase letters indicate significant differences (*p* < 0.05) among cadmium stress concentrations under the same MT concentration, while different lowercase letters denote significant differences (*p* < 0.05) among MT concentrations under the same cadmium stress concentration.

**Figure 7:**
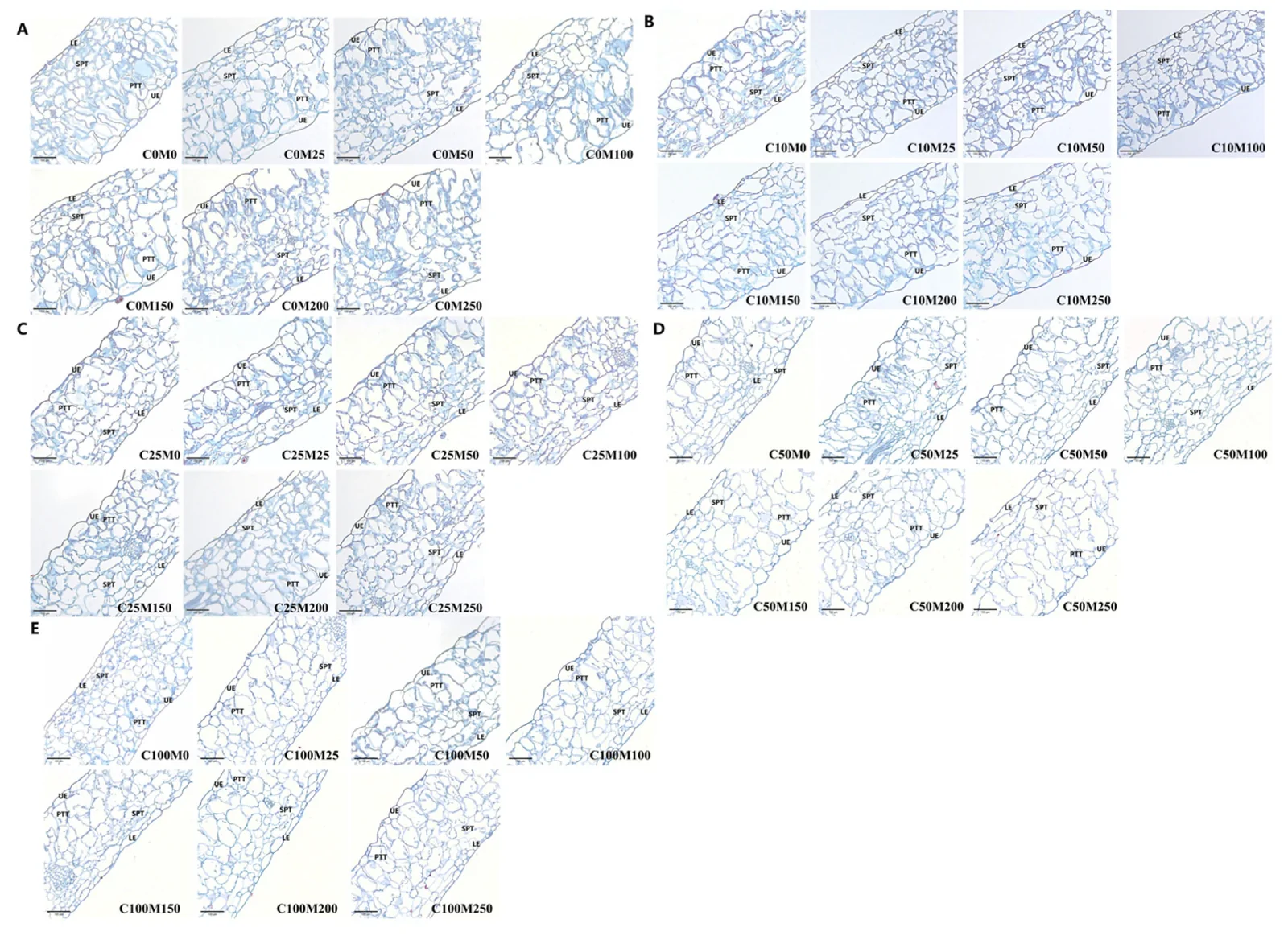
Leaf anatomical structures of *P. fengdouensis* seedlings under exogenous MT and Cd stress. (**A**): Leaf anatomical structure under 0 mg·kg^−1^ Cd stress with different MT concentrations; (**B**): 10 mg·kg^−1^ Cd; (**C**): 25 mg·kg^−1^ Cd; (**D**): 50 mg·kg^−1^ Cd; (**E**): 100 mg·kg^−1^ Cd. Note: Within each Cd level, the seven images correspond to 0, 25, 50, 100, 150, 200, and 250 μM exogenous MT. “UE” represents upper epidermal cells, “LE” represents lower epidermal cells, “PTT” represents palisade tissue, and “SPT” represents spongy tissue.

Increasing Cd stress significantly reduced leaf area, circumference, length, and width of *P. fengdouensis* seedlings, with the smallest values at 100 mg/kg Cd (Figure 5A–F). No significant differences in leaf morphology were found between 50 and 100 mg/kg Cd treatments. Exogenous MT at ≥150 μM enhanced leaf development, with peak values declining at higher concentrations. Without Cd, 200 μM MT maximized leaf area and width, while 150 μM MT optimized circumference and length. MT most effectively alleviated Cd stress at 10 mg/kg Cd. Under 150–200 μM MT, all leaf parameters exceeded control levels. At 25–100 mg/kg Cd, 200 μM MT significantly increased leaf area and length. No significant differences occurred in leaf shape or functional indices. In conclusion, Cd stress inhibited seedling growth by impairing leaf morphology, which was effectively mitigated by MT, particularly at 150–200 μM. Cd stress minimized leaf tissue thickness in *P. fengdouensis* seedlings at 100 mg/kg Cd. Exogenous MT reduces damage: thickness peaks at 200 μM for low-medium Cd and 250 μM for high Cd. No significant differences occur between 200 and 250 μM MT across lower epidermis, palisade, or spongy tissues. Cd stress disrupts leaf integrity and inhibits growth; MT restores thickness to mitigate damage, with 200 μM as optimal (Figure 6A–E). Representative leaf anatomical structures under the different Cd and MT treatment combinations are shown in Figure 7.

**Figure 8:**
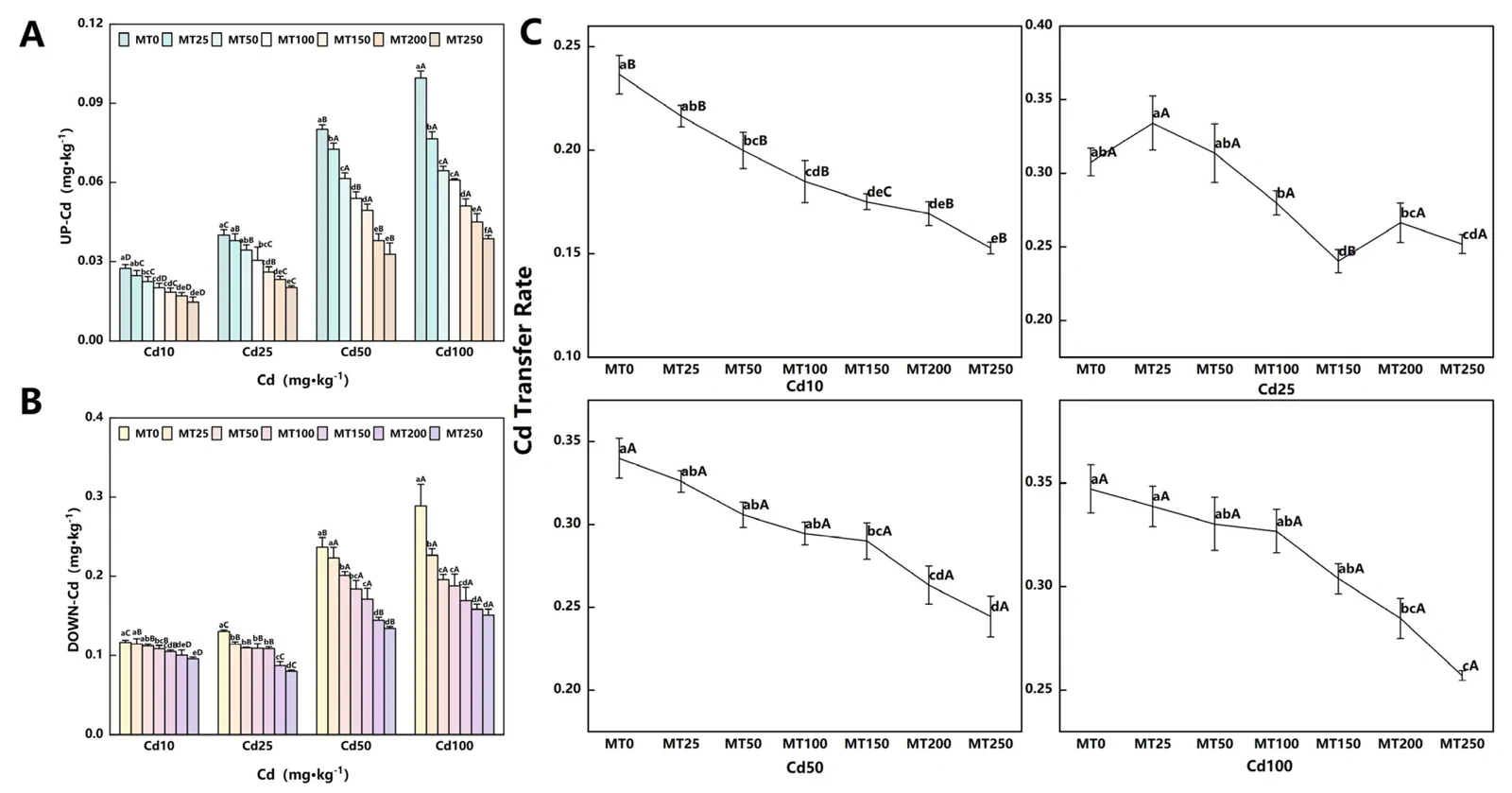
Effects of exogenous MT on Cd accumulation and root-to-shoot translocation in *P. fengdouensis* seedlings. (**A**): Cd content in shoots; (**B**): Cd content in roots; (**C**): Cd translocation factor (shoot Cd/root Cd). In panel (**C**), the four subpanels from upper left to lower right correspond to Cd10, Cd25, Cd50, and Cd100, respectively. Cd concentrations are expressed as mg Cd kg^−1^ dry substrate. MT0-MT250 denote 0–250 μM MT. Different uppercase letters indicate significant differences (*p* < 0.05) among Cd concentrations at the same MT concentration, and different lowercase letters indicate significant differences among MT concentrations at the same Cd concentration.

Cd exposure increased Cd accumulation in *P. fengdouensis* seedlings (Figure 8A,B). At 100 mg kg^−1^ Cd, shoot and root Cd contents reached 0.10 and 0.29 mg kg^−1^ dry biomass, respectively, representing increases of 261.63% and 148.17% relative to the corresponding control values. MT significantly lowered Cd concentrations in both organs (*p* < 0.05) in a generally dose-dependent manner. At 250 μM MT, shoot Cd content decreased by 46.82–61.11% and root Cd content by 17.73–47.74% across the Cd treatments; 200 and 250 μM MT were not significantly different in several comparisons. The Cd translocation factor, calculated as shoot Cd/root Cd, increased with Cd exposure and reached 0.35 at 100 mg kg^−1^ Cd (Figure 8C). MT reduced the translocation factor by up to 35.40%, 21.87%, 28.05%, and 25.96% under 10, 25, 50, and 100 mg kg^−1^ Cd, respectively. Thus, the data show lower Cd accumulation and lower root-to-shoot translocation after MT treatment; they do not by themselves identify the specific uptake or transport mechanism.

### 2.3. Effects of Exogenous MT on the Physiological Traits of P. fengdouensis Seedlings Under Cd Stress

**Figure 9:**
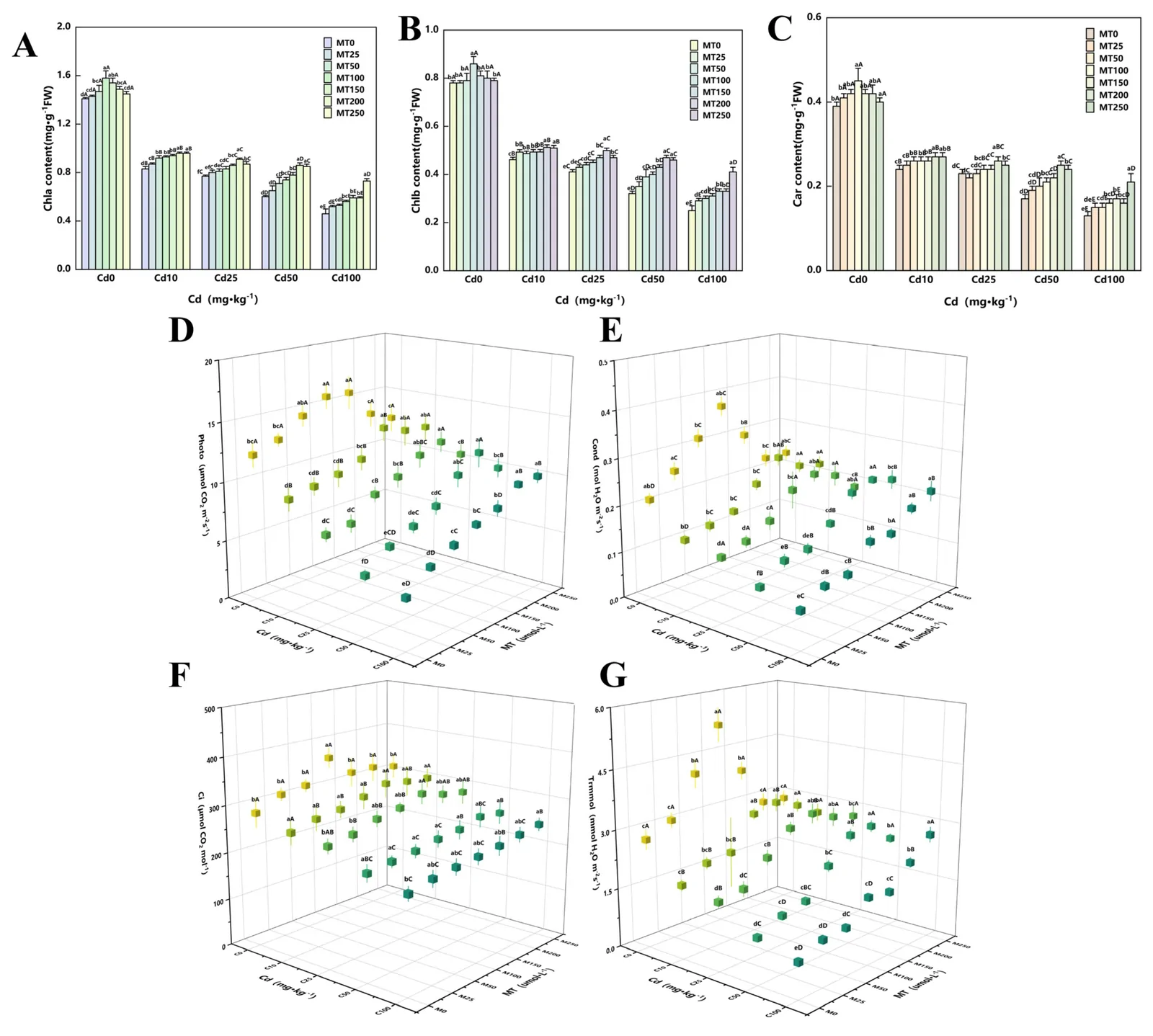
Effects of exogenous MT on photosynthetic pigments and gas-exchange traits of *P. fengdouensis* seedlings under Cd stress. Note: (**A**). Chlorophyll a content; (**B**). Chlorophyll b content; (**C**). Carotenoid content; (**D**). Net photosynthetic rate; (**E**). Stomatal conductance; (**F**). Intercellular CO_2_ concentration; (**G**). Transpiration rate.

Cd stress significantly inhibited photosynthetic performance in *P. fengdouensis* seedlings. Chlorophyll a, chlorophyll b, and carotenoid contents declined progressively with increasing Cd concentration and reached their lowest values at 100 mg/kg Cd, decreasing by 67.12%, 67.25%, and 65.68%, respectively, relative to C0M0. Exogenous MT significantly increased photosynthetic pigment contents under Cd stress (*p* < 0.05). Except under 100 mg/kg Cd, pigment contents generally increased and then decreased with increasing MT concentration; the highest values occurred at 100 μM MT under 10 mg/kg Cd and at 200 μM MT under 25 and 50 mg/kg Cd. Under 100 mg/kg Cd, pigment contents increased continuously with MT concentration and peaked at 250 μM MT, with chlorophyll a, chlorophyll b, and carotenoids increasing by 58.80%, 60.32%, and 54.14%, respectively, compared with C100M0 (Figure 9A–C). Cd stress also reduced net photosynthetic rate (Pn), stomatal conductance (Cond), intercellular CO2 concentration (Ci), and transpiration rate (Tr), whereas exogenous MT alleviated these reductions. For most Cd treatments, the gas-exchange parameters showed an increase followed by a decrease as MT concentration increased. The strongest response under 10 mg/kg Cd generally occurred at 150 μM MT, whereas 200 μM MT produced the best overall mitigation across the Cd treatments; under 100 mg/kg Cd, the parameters continued to increase and generally reached their highest values at 250 μM MT (Figure 9D–G). Overall, exogenous MT at approximately 200–250 μM was most effective in maintaining photosynthetic function under Cd stress.

**Figure 10:**
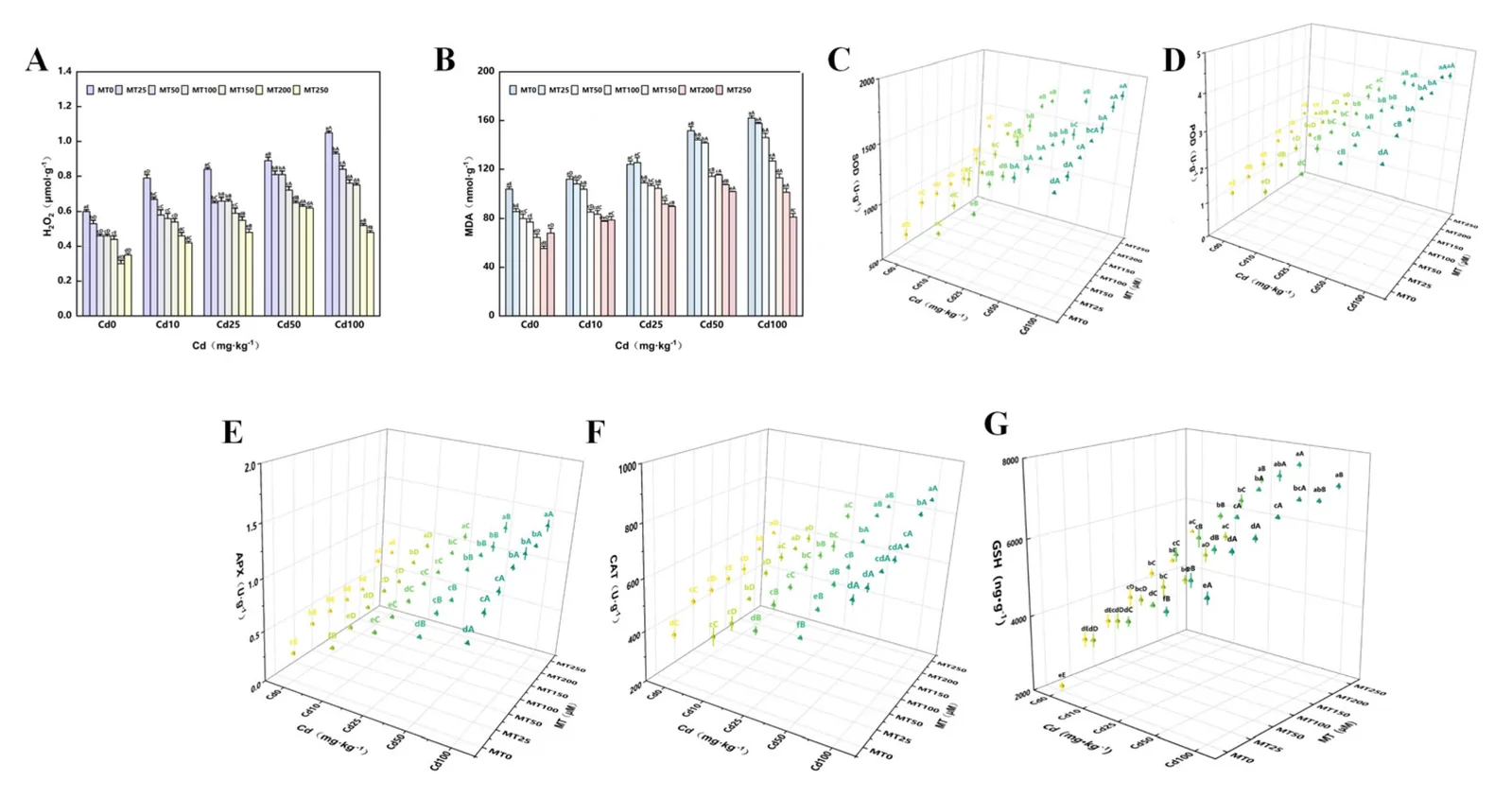
Effects of exogenous MT on oxidative damage and antioxidant defense in *P. fengdouensis* seedlings under Cd stress. Note: (**A**). Hydrogen peroxide (H_2_O_2_) content; (**B**). Malondialdehyde (MDA) content; (**C**). Superoxide dismutase (SOD) activity; (**D**). Peroxidase (POD) activity; (**E**). Catalase (CAT) activity; (**F**). Ascorbate peroxidase (APX) activity; (**G**). Glutathione (GSH) content.

Cd stress promoted oxidative damage in *P. fengdouensis* seedlings, as indicated by progressive increases in H_2_O_2_ and MDA, both of which were highest at 100 mg kg^−1^ Cd. Exogenous MT significantly lowered H_2_O_2_ and MDA contents (*p* < 0.05), with the greatest reductions generally observed at 200–250 μM MT (Figure 10A,B). These lower oxidative-damage markers are consistent with improved redox protection but do not, by themselves, demonstrate direct chemical scavenging by MT. Antioxidant enzymes responded strongly to Cd stress: SOD activity first increased and then decreased, whereas POD, CAT, and APX generally increased with Cd concentration. MT further enhanced the activities of these enzymes, with the strongest effects typically at 200 or 250 μM (Figure 10C–F). GSH, a major non-enzymatic antioxidant and precursor for phytochelatin biosynthesis, also increased under Cd and was further promoted by MT (Figure 10G). Because phytochelatins were not measured, any contribution of the GSH-phytochelatin pathway remains a hypothesis rather than a direct result of this study.

**Figure 11:**
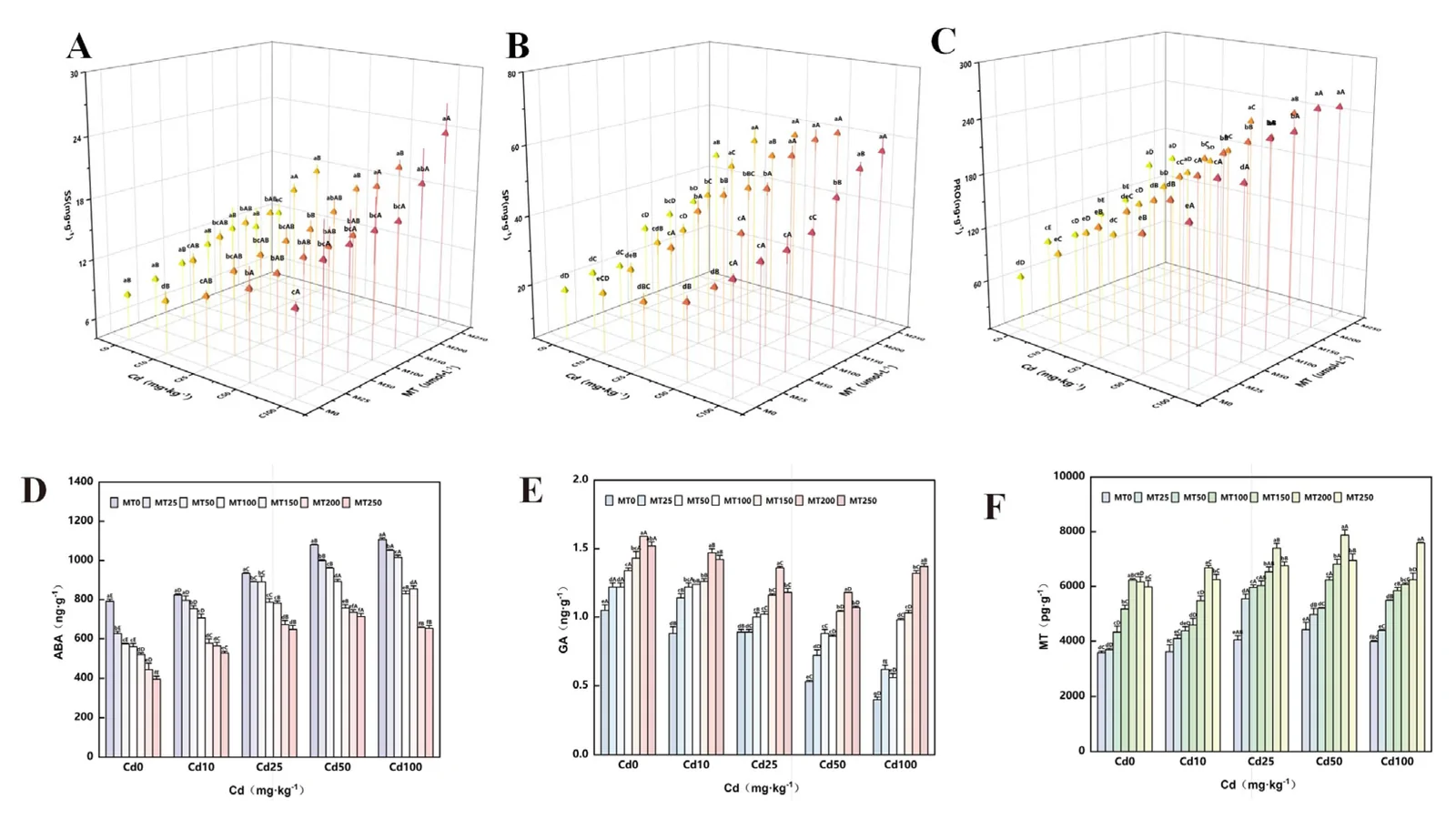
Effects of exogenous MT on osmotic adjustment and endogenous hormone levels in *P. fengdouensis* seedlings under Cd stress. Note: (**A**). Soluble sugar (SS) content; (**B**). Soluble protein (SP) content; (**C**). Free proline (Pro) content; (**D**). Abscisic acid (ABA) content; (**E**). Gibberellin (GA) content; (**F**). Endogenous melatonin (MT) content. Different uppercase letters indicate significant differences (*p* < 0.05) among cadmium stress concentrations under the same MT concentration, while different lowercase letters denote significant differences (*p* < 0.05) among MT concentrations under the same cadmium stress concentration.

Cd stress also altered osmotic adjustment and endogenous hormone levels. Soluble sugar (SS) content increased under Cd stress and peaked at 50 mg/kg Cd, whereas soluble protein (SP) and free proline (Pro) contents increased with Cd concentration and reached their highest levels at 100 mg/kg Cd. Exogenous MT further promoted the accumulation of SS, SP, and Pro; the strongest effects were generally observed at 200–250 μM MT, and differences between 200 and 250 μM MT were not significant under several Cd treatments (*p* > 0.05) (Figure 11A–C). Endogenous ABA increased with Cd concentration and reached its maximum at 100 mg/kg Cd, whereas endogenous GA decreased and reached its minimum at the same Cd level. Endogenous MT increased under Cd stress and peaked at 50 mg/kg Cd. Exogenous MT significantly reduced ABA accumulation and increased GA and endogenous MT levels (*p* < 0.05). ABA was lowest at 250 μM exogenous MT across Cd treatments. GA peaked at 200 μM MT under 0–50 mg/kg Cd and at 250 μM MT under 100 mg/kg Cd. Endogenous MT peaked at 150 μM exogenous MT without Cd, at 200 μM under 10–50 mg/kg Cd, and at 250 μM under 100 mg/kg Cd (Figure 11D–F). Collectively, these results suggest that 200–250 μM exogenous MT improves osmotic adjustment and helps restore hormonal homeostasis under Cd stress.

### 2.4. Leaf Transcriptome Profiling and Co-Expression Responses to Cd Stress and Exogenous MT

**Figure 12:**
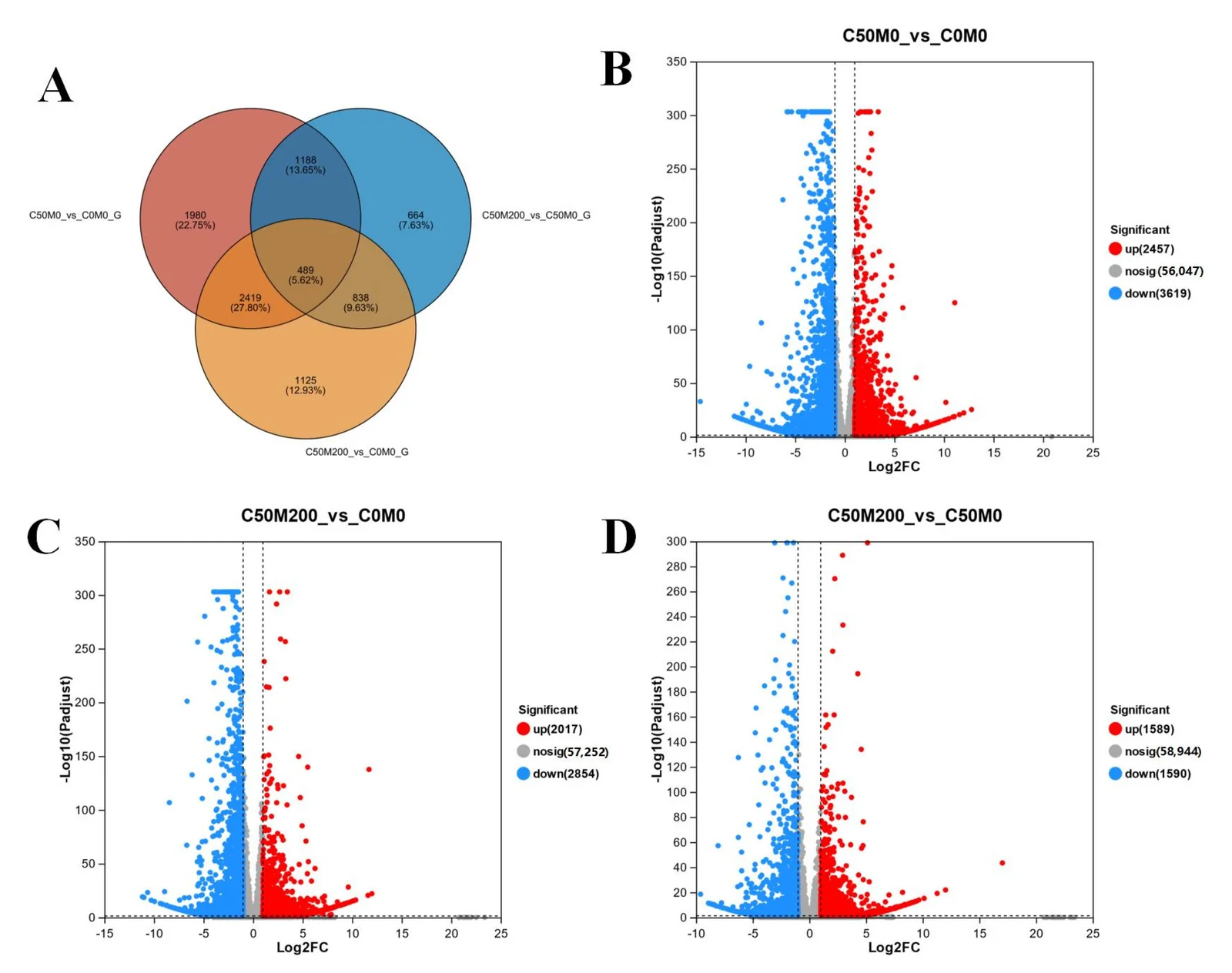
Differentially expressed genes among *P. fengdouensis* treatment groups. Note: (**A**). Venn diagram of DEGs among treatment comparisons; (**B**). Differential expression analysis of C50M0 vs. C0M0; (**C**). Differential expression analysis of C50M200 vs C0M0; (**D**). Differential expression analysis of C50M200 vs. C50M0. The dotted lines in Panels (**B**–**D**) denote the cut-off criteria for differential expression analysis. Points above the horizontal line and to the right of the right vertical line are significantly up-regulated features; points above the horizontal line and to the left of the left vertical line are significantly down-regulated features.

De novo assembly of the nine leaf RNA-seq libraries generated 62,123 unigenes and 125,217 transcripts, consistent with the original assembly statistics. For downstream gene-level analyses, the longest transcript per gene cluster was retained as the representative unigene. Differentially expressed genes (DEGs) were identified with DESeq2 using |log_2_FC| ≥ 1 and adjusted *p* < 0.05. Pairwise comparisons identified 6076 DEGs in C50M0 vs. C0M0 (2457 upregulated and 3619 downregulated), 4871 DEGs in C50M200 vs. C50M0 (2017 upregulated and 2854 downregulated), and 3179 DEGs in C50M200 vs. C0M0 (1589 upregulated and 1590 downregulated). C50M0 vs. C0M0 therefore showed the largest transcriptional response, whereas C50M200 vs. C0M0 showed the fewest DEGs (Figure 12A–D). No tandem-mass-tag (TMT) proteomic analysis was performed in this study.

**Figure 13:**
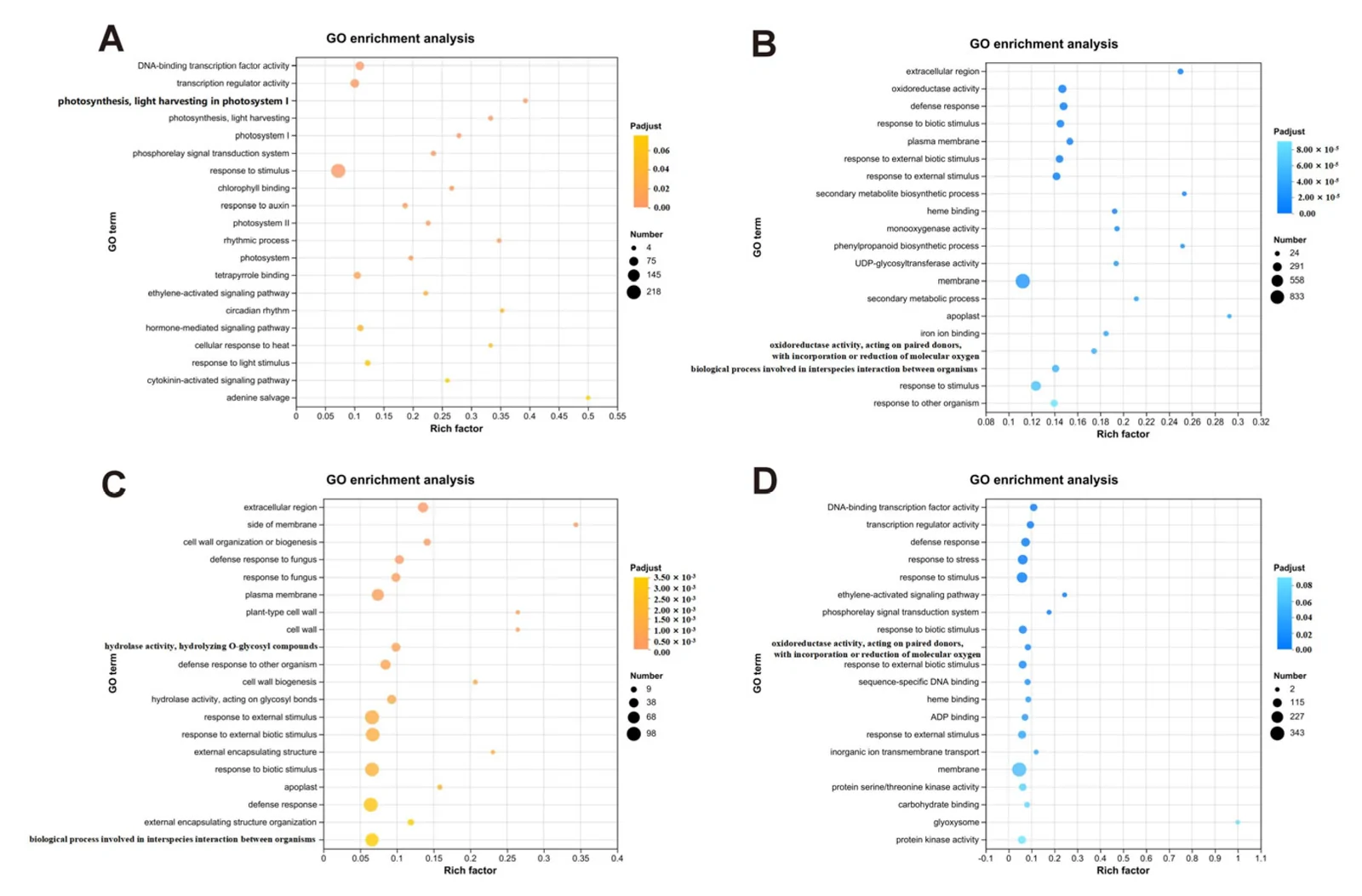
GO enrichment of DEGs in *P. fengdouensis* under Cd stress and exogenous MT treatment. Note: (**A**). Upregulated DEGs in C50M0 vs. C0M0; (**B**). Downregulated DEGs in C50M0 vs. C0M0; (**C**). Upregulated DEGs in C50M200 vs. C50M0; (**D**). Downregulated DEGs in C50M200 vs. C50M0. In (**A**–**D**), the x-axis represents the enrichment factor; bubble size represents the number of DEGs, and color intensity represents enrichment significance.

Gene Ontology (GO) enrichment analysis showed distinct functional responses to Cd stress and MT treatment. In C50M0 vs. C0M0, upregulated DEGs were significantly enriched in 13 biological process (BP), four molecular function (MF), and three cellular component (CC) categories. Major BP terms included photosynthesis and light harvesting, response to light stimulus, auxin and hormone-mediated signaling, cytokinin-activated signaling, rhythmic processes, circadian rhythm, and cellular response to heat. Major MF terms included DNA-binding transcription factor activity, transcription regulator activity, chlorophyll binding, and tetrapyrrole binding, whereas the principal CC terms were photosystem I, photosystem II, and photosystem (Figure 13A). Downregulated DEGs were enriched in defense response, phenylpropanoid biosynthesis, secondary metabolism, extracellular region, plasma membrane, apoplast, oxidoreductase activity, heme binding, monooxygenase activity, UDP-glycosyltransferase activity, and iron ion binding (Figure 13B). In C50M200 vs. C50M0, upregulated DEGs were enriched particularly in cell-wall organization or biogenesis, defense responses, extracellular region, plasma membrane, plant-type cell wall, and glycosyl hydrolase activity (Figure 13C), whereas downregulated DEGs included stress responses, ethylene-activated signaling, inorganic ion transmembrane transport, oxidoreductase activity, sequence-specific DNA binding, and protein kinase activity (Figure 13D). These data support MT-associated remodeling of cell-wall/plasma-membrane and stress-response functions.

**Figure 14:**
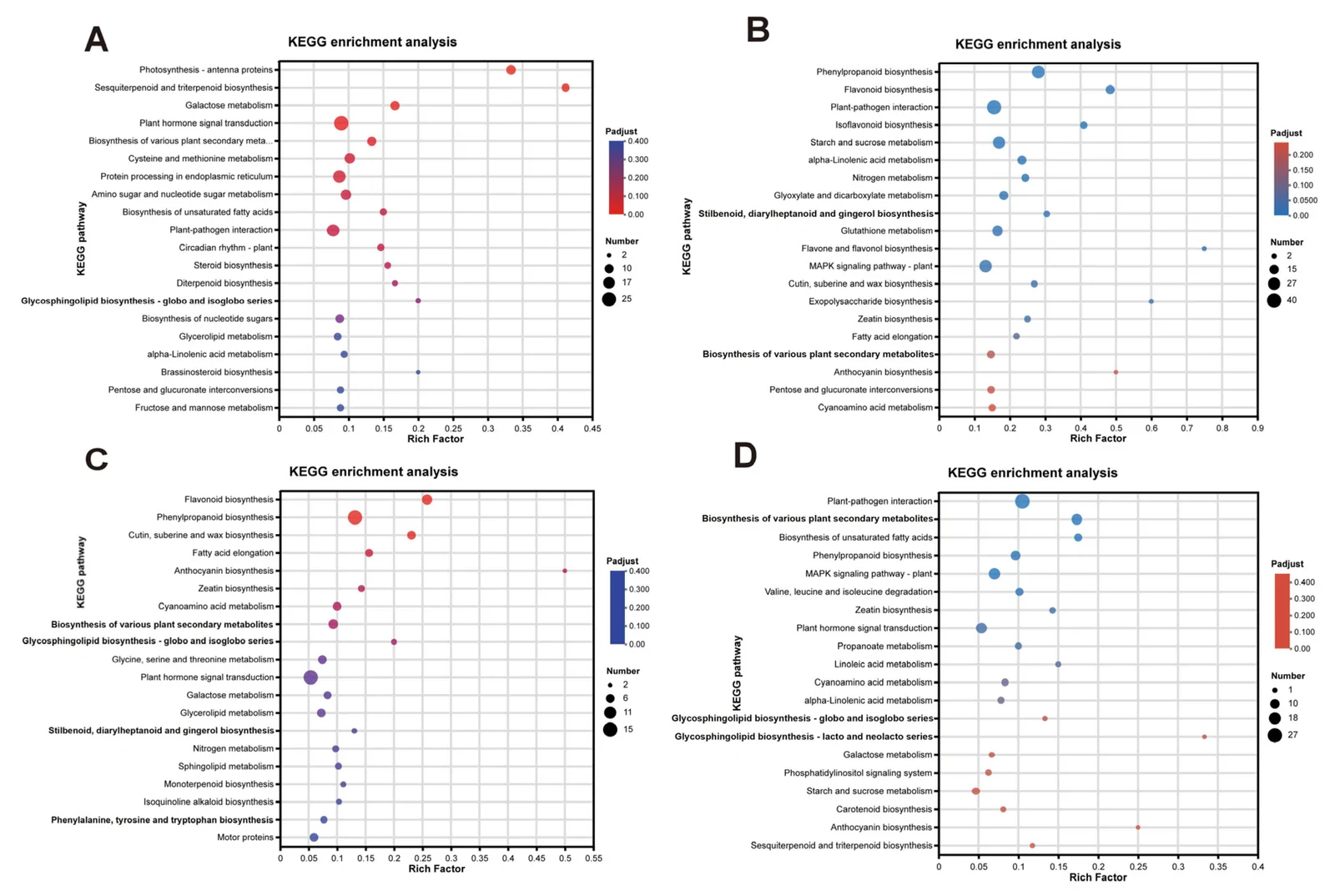
KEGG enrichment of DEGs in *P. fengdouensis* under Cd stress and exogenous MT treatment. Note: (**A**). Upregulated DEGs in C50M0 vs. C0M0; (**B**). Downregulated DEGs in C50M0 vs. C0M0; (**C**). Upregulated DEGs in C50M200 vs. C50M0; (**D**). Downregulated DEGs in C50M200 vs. C50M0. In (**A**–**D**), the x-axis represents the enrichment factor; bubble size represents the number of DEGs, and color intensity represents enrichment significance.

Kyoto Encyclopedia of Genes and Genomes (KEGG) enrichment further revealed extensive metabolic and signaling reprogramming. In C50M0 vs. C0M0, representative enriched pathways included plant hormone signal transduction, plant-pathogen interaction, circadian rhythm, alpha-linolenic acid metabolism, glycerolipid metabolism, and protein processing in the endoplasmic reticulum (Figure 14A), whereas downregulated DEGs were enriched in MAPK signaling, phenylpropanoid and flavonoid biosynthesis, starch and sucrose metabolism, nitrogen metabolism, and glutathione metabolism (Figure 14B). In C50M200 vs. C50M0, upregulated DEGs were enriched in plant hormone signal transduction, motor proteins, flavonoid and phenylpropanoid biosynthesis, cutin/suberin/wax biosynthesis, anthocyanin biosynthesis, and amino-acid-related metabolism (Figure 14C). Downregulated DEGs included plant-pathogen interaction, MAPK signaling, and plant hormone signal transduction (Figure 14D). These enrichment patterns are consistent with MT-associated changes in antioxidant secondary metabolism, protective surface/cell-wall functions, and stress signaling, but pathway enrichment alone does not establish causal activation of a specific mechanism.

**Figure 15:**
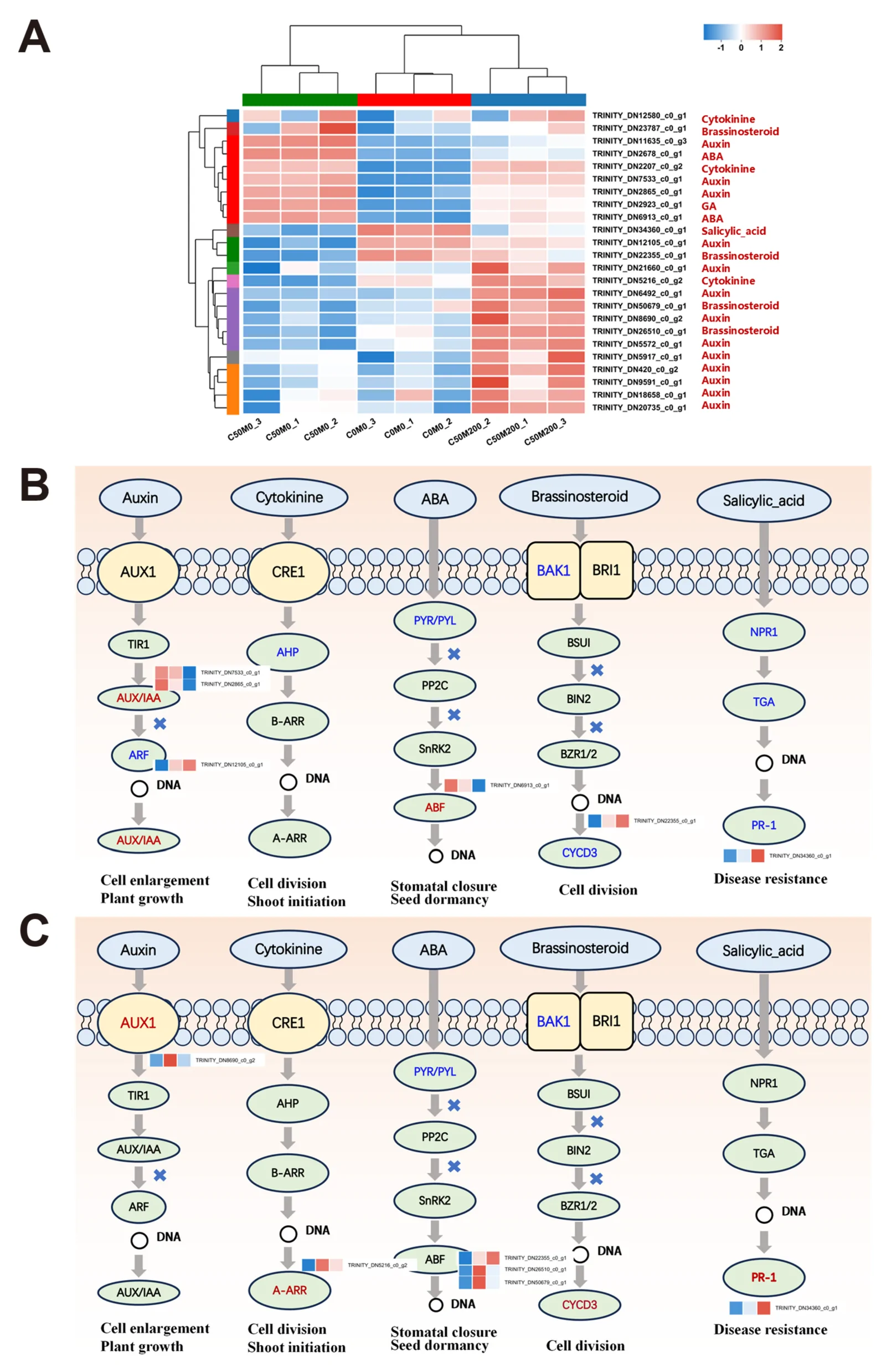
Integrated analysis of plant hormone signaling under Cd stress and exogenous MT treatment in *P. fengdouensis*. Note: (**A**). Clustering of plant hormone signaling genes; (**B**). Hormone pathways under Cd stress; (**C**). Remodeling of plant hormone signaling by exogenous MT under Cd stress.

Plant hormone signal transduction was a prominent component of the MT-associated transcriptomic response. Among the top 20 KEGG pathways enriched with upregulated DEGs in C50M200 vs. C50M0, five were directly or indirectly hormone-related, and nine of the top 20 pathways enriched with downregulated DEGs were hormone-related. The expression data showed changes in auxin-, cytokinin-, gibberellin-, ABA-, brassinosteroid-, and salicylic-acid-related genes (Figure 15A–C). Specifically, Cd stress was associated with higher AUX/IAA expression, whereas MT treatment increased AUX1 and reduced GH3 expression; Cd suppressed AHP, whereas MT increased A-ARR; and MT-associated changes in PYR/PYL occurred in parallel with the measured decline in endogenous ABA. MT also restored CYCD3 and PR-1 expression relative to Cd stress. These observations demonstrate treatment-associated expression remodeling. Functional consequences such as altered auxin transport, cytokinin feedback, or salicylic-acid defense are biologically plausible interpretations that require targeted validation.

**Figure 16:**
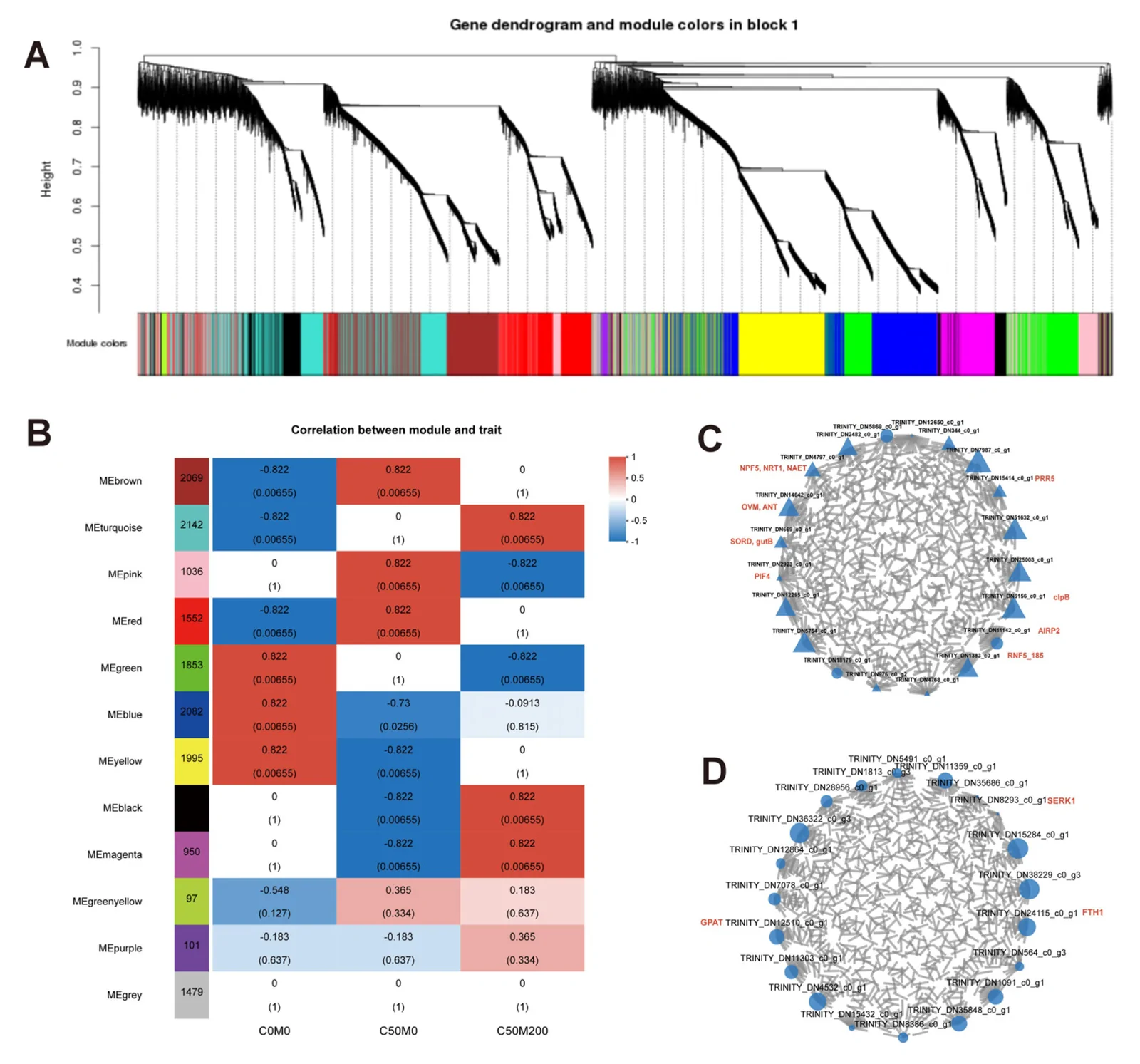
Exploratory weighted gene co-expression network analysis (WGCNA) under Cd stress and exogenous MT treatment in *P. fengdouensis*. (**A**): Gene clustering dendrogram and module assignment; (**B**): module eigengene correlations with the three treatment-indicator traits C0M0, C50M0, and C50M200 (values are Pearson correlation coefficients with *p* values in parentheses; numbers at the left indicate module gene counts); (**C**): candidate core-gene network associated with Cd stress; (**D**): candidate core-gene network associated with exogenous MT treatment. WGCNA was based on nine transcriptomes and is interpreted as hypothesis-generating.

Exploratory weighted gene co-expression network analysis (WGCNA) of the nine leaf transcriptomes identified 12 co-expression modules (Figure 16A). The network was constructed using a soft-thresholding power β = 3, minimum module size = 30, minimum KME to stay = 0.3, and merge cut height = 0.25. The columns in the module-trait heat map (Figure 16B) are treatment-indicator traits for C0M0, C50M0, and C50M200; each cell reports the module eigengene-treatment correlation and its *p* value, and the number shown at the left of each row is the number of genes in that module. Module membership (MM) denotes the correlation between a gene and its module eigengene, whereas gene significance (GS) denotes association between a gene and the treatment trait. Cd-associated genes were concentrated mainly in Brown, Pink, and Red modules, whereas MT-associated genes were concentrated mainly in Turquoise, Black, and Magenta modules (*p* < 0.01). Candidate networks included clpB1, RNF5_185, AIRP2, PRR5, PIF4, FTH1, GPAT, and a MADS-box transcription factor (Figure 16C,D). Given the small sample size (three treatments × three biological replicates), module stability and hub-gene assignments are considered exploratory rather than definitive.

## 3. Discussion

### 3.1. Exogenous MT Alleviates Cd Effects on Seed Germination and Early Seedling Development

Seed germination is a critical stage for the persistence of the narrowly distributed *P. fengdouensis*, and our data show a clear decline in germination potential and germination rate as Cd concentration increased. This pattern is consistent with reports for cotton and wheat under Cd stress [31,32]. At 50 mg L^−1^ Cd, early seedling root, stem, leaf, biomass, and pigment traits were also suppressed, indicating that Cd effects extended beyond radicle emergence. Exogenous MT, especially at 100 μM, restored multiple germination and early-growth traits, consistent with MT-mediated alleviation reported in cotton and pea [31,33]. The present experiment did not directly track MT entry into seeds or quantify intracellular MT during imbibition; therefore, statements that MT “permeates seeds” have been removed. Instead, the data support an association between exogenous MT treatment and improved germination/early seedling performance, while antioxidant, osmotic, and metal-homeostasis mechanisms are discussed as literature-supported interpretations rather than directly demonstrated processes.

### 3.2. Exogenous MT Modulates Seedling Growth, Morphology, and Cd Accumulation/Translocation

Cd stress significantly inhibited seedling height, biomass accumulation, root development, leaf expansion, and leaf anatomical thickness in *P. fengdouensis*, with the strongest effects generally occurring at 50–100 mg kg^−1^ Cd. Exogenous MT partly reversed these responses, particularly at 150–250 μM. The root-growth response is consistent with the broader involvement of auxin signaling in Cd-stressed roots and with our leaf transcriptome showing MT-associated changes in AUX1 and GH3, but the present study did not measure PIN proteins or nitric oxide (NO); the previous unsupported PIN/NO mechanism has therefore been removed. Cd accumulated more strongly in roots than shoots, and MT reduced both organ Cd concentrations and the shoot/root Cd translocation factor. This pattern agrees with studies in which MT reduced root-to-shoot Cd movement [34,35]. Because neither Cd influx kinetics nor xylem loading was measured directly, the results are described as reduced Cd accumulation and translocation rather than proof of a specific uptake-blocking mechanism.

### 3.3. Exogenous MT: P. fengdouensis Seedlings (Physiol. Traits) Under Cd Stress

Cd stress reduced photosynthetic pigments and gas-exchange parameters in *P. fengdouensis*, whereas MT partly restored these traits. The literature suggests that Cd can disrupt chlorophyll metabolism and stomatal function, and MT can help maintain photosynthetic performance [36,37,38,39]; however, these mechanisms were not directly assayed here. Cd also increased H_2_O_2_ and MDA, while MT treatment was associated with lower concentrations of both markers and higher SOD, POD, CAT, and APX activities. The present data therefore support reduced oxidative damage and strengthened antioxidant defense, but do not demonstrate that MT directly scavenged H_2_O_2_ or peroxyl radicals in vivo. GSH should be interpreted as an important non-enzymatic antioxidant and a precursor for phytochelatin biosynthesis, not as a peroxidase precursor. Although higher GSH could support Cd chelation through phytochelatins, phytochelatins were not measured in this study and this pathway remains inferential. Cd increased ABA and decreased GA, whereas exogenous MT reduced ABA and increased GA over much of the tested range. Changes in stomatal conductance and ABA may influence transpiration-driven root-to-shoot Cd translocation; they should not be interpreted as direct evidence that stomatal closure reduces root Cd uptake. Collectively, the physiological data indicate coordinated photosynthetic, antioxidant, osmotic, and hormonal responses to MT, with 200–250 μM generally providing the strongest seedling-level mitigation.

### 3.4. Transcriptomic Responses and Candidate Regulatory Networks Under Cd Stress and MT Treatment

The transcriptome data directly show treatment-associated changes in genes and enriched pathways related to photosynthesis, cellular components such as the plasma membrane and cell wall, secondary metabolism, and hormone signaling. In C50M200 vs. C50M0, enrichment of cell-wall organization, extracellular-region, plasma-membrane, flavonoid/phenylpropanoid, cutin/suberin/wax, and hormone-signaling functions is consistent with a shift toward structural protection, redox regulation, and growth-stress rebalancing. Likewise, the observed changes in AUX1, GH3, A-ARR, PYR/PYL, CYCD3, and PR-1 establish transcriptional responses in several hormone pathways. By contrast, proposed roles such as enhanced Cd chelation by FTH1, membrane stabilization by GPAT, or developmental reprogramming by MADS-box factors are hypotheses based on gene annotation, co-expression patterns, and evidence from other species, not direct functional demonstrations in *P. fengdouensis*. The WGCNA [40] modules similarly provide candidate gene sets rather than causal networks. We therefore distinguish throughout this section between findings measured in *P. fengdouensis* and mechanistic interpretations extrapolated from Arabidopsis, rice, and other model systems.

### 3.5. Limitations and Scope of Inference

Several limitations should be considered when interpreting this study. First, transcriptomes were obtained from leaves because the physiological endpoints most strongly affected by MT (photosynthesis, oxidative status, leaf anatomy, and endogenous hormones) were measured in leaf tissue and MT was applied foliarly; these data therefore represent the leaf response and do not substitute for root transcriptomics of Cd uptake. Second, the WGCNA [40] included only nine samples and is used here as an exploratory tool; module stability should be verified with a larger dataset. Third, qRT-PCR validation and functional assays of candidate genes were not included in the original experimental design, so transcriptome-derived mechanisms remain candidates. Fourth, Cd subcellular distribution and phytochelatin concentrations were not measured. Finally, some methodological metadata from the completed experiment, including substrate pH, the exact MT spray volume/interval, LI-6400 chamber set points, and ELISA kit recovery information, were not retained in the archived records and therefore cannot be reconstructed without speculation.

## 4. Materials and Methods

### 4.1. Seed Germination and Early Seedling Development Experiment Under Cd Stress and Exogenous MT

Mature *P. fengdouensis* seeds were collected in April 2023 from the Deganba riverbank in Jiangjin District, Chongqing (29°14′30.89″ N, 106°16′8.18″ E) and stored sealed at 4 °C. Five nominal Cd concentrations (0, 10, 25, 50, and 100 mg L^−1^ Cd in aqueous solution, prepared with CdCl_2_·2.5H_2_O) were first tested. Based on the germination response, 50 mg L^−1^ Cd was selected for the subsequent MT concentration experiment. Seven MT concentrations were used: 0, 25, 50, 100, 150, 200, and 250 μM (Table 1). Plump, size-matched seeds were soaked for 8 h in sterile distilled water or the assigned MT solution, disinfected for 5 min in 75% ethanol, rinsed twice with distilled water, disinfected for 5 min in 0.1% NaClO, and thoroughly rinsed again. Seeds were then placed in Petri dishes lined with two layers of filter paper and supplied with 10 mL distilled water or the assigned treatment solution. The growth chamber was maintained at 25/20 °C day/night, 60–70% relative humidity, 62.5 μmol m^−2^ s^−1^ irradiance, and a 12 h photoperiod. Each treatment comprised three Petri dishes with 30 seeds per dish and was followed for 7 d while the filter paper was kept moist.

Germination potential (GP) was calculated as the percentage of seeds germinated by day 3; germination rate (GR) as the percentage germinated by day 7; germination index (GI) as Σ(ni/di), where ni is the number of newly germinated seeds on day i and di is the corresponding day; and seed vigor index (SVI) as GI × total root length. At day 7, organ lengths were measured on three randomly selected seedlings per treatment, and fresh/dry biomass was determined from 20 seedlings per treatment. Photosynthetic pigments were extracted from 0.1 g tissue in acetone:ethanol:water (4.5:4.5:1) for 48 h in darkness and quantified spectrophotometrically at 663, 645, and 470 nm. These germination indices and seedling vigor measurements were calculated following established seed testing procedures and vigor assessment protocols [41,42,43].

### 4.2. Seedling Growth, Morphology, Anatomy, and Cd Accumulation Experiment

Seedlings derived from the germination experiment were transferred to pots containing a 3:1:1 mixture of nutrient soil:vermiculite:perlite and pre-cultivated for four months under the chamber conditions described above, with periodic application of Hoagland nutrient solution. Uniform, healthy seedlings (210 in total) were then assigned to a 5 × 7 factorial design comprising five Cd levels (0, 10, 25, 50, and 100 mg Cd kg^−1^ substrate dry mass) and seven MT levels (0, 25, 50, 100, 150, 200, and 250 μM), with six biological replicates per treatment (Table 2). Each pot contained 200 g of substrate (dry-mass basis). CdCl_2_·2.5H_2_O was mixed thoroughly into the substrate to provide 0, 2, 5, 10, or 20 mg elemental Cd per pot, corresponding to approximately 0, 4.063, 10.157, 20.314, or 40.628 mg CdCl_2_·2.5H_2_O per pot, respectively. The amended substrate was equilibrated for 30 d before transplanting. No additional Cd solution was applied after transplanting; Cd exposure continued through the pre-amended substrate. MT was applied to leaves in equal-volume foliar treatments at regular intervals for 15 d, with distilled water used for the MT0 controls. The archived experimental record specifies the 15 d treatment duration but does not retain the exact spray volume or interval.

At the end of the 15 d treatment, three plants per treatment were randomly selected for measurements of plant height, biomass, leaf morphology, root morphology, and leaf anatomy. Plants were washed with 2 mM EDTA-Na_2_ for 5 min and then with ultrapure water before separation into shoots and roots. Biomass samples were dried at 105 °C for 30 min and then at 80 °C to constant mass. Leaf area, perimeter, length, and width were measured using a CI-203 leaf-area meter; root length, surface area, and volume were quantified from digital scans. For anatomy, 0.5 × 0.5 cm pieces from the middle of fully expanded leaves (including the main vein) were fixed in formaldehyde:glacial acetic acid:70% ethanol (5:5:90, *v*/*v*/*v*; tissue:fixative 1:20), prepared following standard plant microtechnique, and measured using NIS-Elements BR. Shoot and root Cd were quantified after HNO_3_-H_2_O_2_ (6:2, *v*/*v*) microwave digestion by flame atomic absorption spectrophotometry; the translocation factor was calculated as shoot Cd/root Cd.

### 4.3. Physiological and Biochemical Measurements of P. fengdouensis Seedlings

Physiological and biochemical measurements were made at the end of the 15 d Cd/MT treatment. For gas exchange, three biological replicates per treatment were measured on leaves from the same developmental position, using the middle portion of the blade while avoiding the main vein. Net photosynthetic rate (Pn), transpiration rate (Tr), intercellular CO_2_ concentration (Ci), and stomatal conductance (Cond) were recorded with an LI-6400 portable photosynthesis system. The archived instrument log does not retain the chamber irradiance, reference CO_2_ concentration, or block-temperature set points; these values are therefore not reconstructed here. Photosynthetic pigments were determined by the direct-extraction method described in Section 4.1. MDA was determined by thiobarbituric-acid colorimetry and H_2_O_2_ spectrophotometrically. SOD was assayed by NBT photoreduction, POD by the guaiacol method, CAT using a commercial assay kit, APX spectrophotometrically, and GSH by Ellman’s method. Proline, soluble protein, and soluble sugar were measured by standard colorimetric methods. Endogenous GA, ABA, and MT were measured with commercial ELISA kits and quantified against assay-specific standard curves. Manufacturer/catalog and recovery metadata were not retained in the archived experimental record and are therefore reported as a limitation rather than inferred. The physiological and biochemical assays were performed according to previously established procedures for pigment determination, lipid peroxidation, reactive oxygen species quantification, and antioxidant enzyme activity measurements [44,45,46,47].

### 4.4. Leaf Transcriptome Sequencing and Bioinformatic Analysis

Based on the morphology and physiology screening, three treatments were selected for RNA-seq: C0M0, C50M0, and C50M200. Three biological replicates (one seedling per replicate) were sampled from each treatment, giving nine libraries. Leaves were chosen because the study quantified photosynthesis, leaf anatomy, oxidative status, and endogenous hormones in leaf tissue and because MT was applied foliarly; therefore, the transcriptome represents the leaf response and should not be interpreted as a direct transcriptomic analysis of root Cd uptake. Leaves from the same developmental position were collected, immediately frozen in liquid nitrogen, and stored at −80 °C.

Total RNA was evaluated with NanoDrop 2000, agarose-gel electrophoresis, and Agilent 5300; library input required ≥1 μg RNA, concentration ≥ 30 ng μL^−1^, RQN > 6.5, and OD260/280 of 1.8–2.2. Poly(A) mRNA was enriched using oligo(dT) beads, fragmented to approximately 300 bp, reverse-transcribed, adapter-ligated, PCR-enriched, and sequenced. Clean reads were assembled de novo with Trinity. Expression was quantified with RSEM. Differential expression was analyzed with DESeq2 [48] using |log_2_FC| ≥ 1 and adjusted *p* < 0.05. Functional annotation/enrichment used NR, Swiss-Prot, Pfam, EggNOG, GO, and KEGG resources. The RNA-seq dataset is deposited in NCBI under accession PRJNA1356312.

WGCNA [40] was performed with soft-thresholding power β = 3, minimum module size = 30, minimum KME to stay = 0.3, and merge cut height = 0.25. Module eigengenes were correlated with treatment-indicator traits (C0M0, C50M0, and C50M200). Module membership (MM) was defined as the correlation between a gene and its module eigengene, and gene significance (GS) as the association between a gene and the treatment trait. Because only nine transcriptomes were available, WGCNA [40] was used for exploratory candidate prioritization. A complete gene-level GS/MM table was not retained in the archived analysis output, so unverified values were not reconstructed. No qRT-PCR validation was included in the original experiment; this is explicitly acknowledged as a limitation.

### 4.5. Statistical Analysis

Statistical analysis was performed using Microsoft Excel 2016 and IBM SPSS Statistics 27. Data are presented as mean ± standard error (SE). For the factorial seedling experiment, Cd and MT effects were evaluated by two-way ANOVA followed by multiple comparisons at *p* < 0.05; the seed-germination screening was analyzed across Cd concentrations and the MT mitigation experiment across MT treatments under the selected Cd concentration. Unless otherwise stated above, biological replicates correspond to independently treated Petri dishes or individual plants. Structural thickness was quantified using Image-Pro Plus 6.0 as described above. Principal component analysis (PCA) was performed in Origin 2024, and figures were prepared with Origin 2024 and Adobe Illustrator 2020.

## 5. Conclusions

Cd stress inhibited germination, seedling growth, photosynthesis, and normal redox/hormonal balance in *P. fengdouensis*. Exogenous MT alleviated these effects in a stage-dependent manner: 100 μM was most effective during germination and early seedling development, whereas 200–250 μM generally produced the strongest responses in four-month-old seedlings. MT treatment was associated with lower shoot/root Cd accumulation and reduced root-to-shoot translocation, lower H_2_O_2_ and MDA, enhanced antioxidant and osmotic-adjustment responses, and shifts in endogenous ABA/GA/MT. Leaf RNA-seq further identified MT-associated changes in cell-wall/plasma-membrane functions, secondary metabolism, and hormone signaling, together with exploratory WGCNA [40] candidate modules. These molecular results identify testable hypotheses rather than proven causal mechanisms because root transcriptomes, qRT-PCR/functional validation, Cd subcellular localization, and phytochelatin measurements were not included. The study therefore provides a physiological and transcriptomic framework for evaluating MT as a potential aid to ex situ conservation and stress management of *P. fengdouensis*, while field applicability and mechanistic causality require further validation.

## Figures and Tables

**Table 1:** Experimental design of seed germination.

Group	Soak the Seed Solution	Petri Dish Add Solution	Measurement Metrics
C0M0	Distilled water	Distilled water 10 mL	Germination vigor, germination rate, germination index, seed vigor index, daily germination count, leaf length, root length, stem length, root-stem ratio, fresh seed weight, dry seed weight, and photosynthetic pigment content
CxM0	Distilled water	Cd solution	
CxM25	25 μM MT solution	5 mL Cd solution + 5 mL MT solution	
CxM50	50 μM MT solution	5 mL Cd solution + 5 mL MT solution	
CxM100	100 μM MT solution	5 mL Cd solution + 5 mL MT solution	
CxM150	150 μM MT solution	5 mL Cd solution + 5 mL MT solution	
CxM200	200 μM MT solution	5 mL Cd solution + 5 mL MT solution	
CxM250	250 μM MT solution	5 mL Cd solution + 5 mL MT solution	

Note: “x” represents the screened Cd concentration (50 mg L^−1^ Cd) used in the MT mitigation experiment.

**Table 2:** Combination of treatment conditions for seedings.

MT Concentration (μM)	Cd Concentration (mg kg^−1^ Dry Substrate)				
	0	10	25	50	100
0	C0M0	C10M0	C25M0	C50M0	C100M0
25	C0M25	C10M25	C25M25	C50M25	C100M25
50	C0M50	C10M50	C25M50	C50M50	C100M50
100	C0M100	C10M100	C25M100	C50M100	C100M100
150	C0M150	C10M150	C25M150	C50M150	C100M150
200	C0M200	C10M200	C25M200	C50M200	C100M200
250	C0M250	C10M250	C25M250	C50M250	C100M250

## Data Availability

The data presented in this study are available on request from the corresponding author due to restrictions related to the protection of endangered wild plant resources.
